# Genetic variation at 11q23.1 confers colorectal cancer risk by dysregulation of colonic tuft cell transcriptional activator POU2AF2

**DOI:** 10.1136/gutjnl-2024-332121

**Published:** 2024-11-28

**Authors:** Vidya Rajasekaran, Bradley T Harris, Ruby T Osborn, Claire Smillie, Kevin Donnelly, Marion Bacou, Edward Esiri-Bloom, Li-Yin Ooi, Morven Allan, Marion Walker, Stuart Reid, Alison Meynert, Graeme Grimes, James P Blackmur, Peter G Vaughan-Shaw, Philip J Law, Ceres Fernández-Rozadilla, Ian Tomlinson, Richard S Houlston, Kevin B Myant, Farhat VN Din, Maria Timofeeva, Malcolm G Dunlop, Susan M Farrington

**Affiliations:** 1 CRUK Edinburgh Centre, Institute of Genetics and Cancer, The University of Edinburgh, Edinburgh, UK; 2 Human Genetics, Wellcome Sanger Institute, Hinxton, UK; 3 The University of Edinburgh MRC Human Genetics Unit, Edinburgh, UK; 4 Department of Pathology, National University of Singapore, Singapore; 5 Division of Genetics and Epidemiology, The Institute of Cancer Research, London, UK; 6 Cancer Predisposition and Biomarkers Lab, Instituto de Investigacion Sanitaria de Santigao de Compostela, Santiago de Compostela, Spain; 7 Department of Oncology, University of Oxford Department of Oncology, Oxford, UK; 8 IST - EBB/Epidemiology, Biostatistics and Biodemography, University of Southern Denmark, Odense, Denmark

**Keywords:** cancer, colorectal cancer, colorectal cancer genes, cancer susceptibility

## Abstract

**Background:**

Common genetic variation at 11q23.1 is associated with colorectal cancer (CRC) risk, exerting local expression quantitative trait locus (cis-eQTL) effects on *POU2AF2*, *COLCA1* and *POU2AF3* genes. However, complex linkage disequilibrium and correlated expression has hindered elucidation of the mechanisms by which genetic variants impart underlying CRC risk.

**Objective:**

Undertake an interdisciplinary approach to understand how variation at 11q23.1 locus imparts CRC risk.

**Design:**

We employ analysis of RNA sequencing, single-cell RNA sequencing, chromatin immunoprecipitation sequencing and single-cell ATAC sequencing data to identify, prioritise and characterise the genes that contribute to CRC risk. We further validate these findings using mouse models and demonstrate parallel effects in human colonic mucosa.

**Results:**

We establish rs3087967 as a prime eQTL variant at 11q23.1, colocalising with CRC risk. Furthermore, rs3087967 influences expression of 21 distant genes, thereby acting as a trans-eQTL hub for a gene-set highly enriched for tuft cell markers. Epigenomic analysis implicates POU2AF2 as controlling the tuft cell-specific trans-genes, through POU2F3-correlated genomic regulation. Immunofluorescence confirms rs3087967 risk genotype (T) to be associated with a tuft cell deficit in the human colon. CRISPR-mediated deletion of the 11q23.1 risk locus genes in the mouse germline exacerbated the *Apc^Min/+^* mouse phenotype on abrogation of *Pou2af2* expression specifically.

**Conclusion:**

We demonstrate that genotype at rs3087967 controls a portfolio of genes through misregulation of *POU2AF2. POU2AF2* is the primary transcriptional activator of tuft cells with a tumour suppressive role in mouse models. We therefore implicate tuft cells as having a key tumour-protective role in the large bowel epithelium.

**WHAT IS ALREADY KNOWN ON THIS TOPIC**

**WHAT THIS STUDY ADDS**

- Several trans-genes are newly implicated as both colonic tuft cell markers and CRC risk genes.
- POU2AF2 is found to mediate both the transcriptional and tumour suppressive effects.

**HOW THIS STUDY MIGHT AFFECT RESEARCH, PRACTICE OR POLICY**

- While the role of tuft cells in the large intestine is ambiguous, their abundance may serve as a novel biomarker of tumourigenic risk in this tissue.
- Further understanding of the pathways impacting tuft cell function and abundance may lead to the development of chemoprevention agents.

## Introduction

 Heritable genetic variation contributes up to 35% of overall colorectal cancer (CRC) risk.^1^ Genome wide-association studies (GWAS) have identified 205 common CRC risk-associated variants, including rs3802842 at 11q23.1^2^ (GRCh38 chr11:110 600 001–112 700 000). CRC-associated variation at 11q23.1 is corroborated by several studies; however, greater risk has been associated with alternative 11q23.1 variants, including rs11213801,^3^ rs3087967^4^, rs7130173 and rs10891245.^5^ High linkage disequilibrium (LD) in this region makes identification of the top-associated variant, the target gene and potential mechanism of dysregulation difficult.

Several studies demonstrate expression quantitative trait loci (eQTL) effects between CRC-associated 11q23.1 variants and three cis-targets*—POU2AF2* and *POU2AF3* (both protein-coding) and *COLCA1* (a long-non-coding RNA).^4 5^ Expression analysis of healthy human colonic mucosa also identified trans-eQTL target genes of rs3087967, a single nucleotide polymorphism (SNP) in the 3’ untranslated region (3’UTR) of *POU2AF2.*^6^ Cell-specific mapping of 11q23.1 trans-eQTL target expression indicates these genes are putative markers of tuft cells and comprise a *POU2AF2*-correlated, tuft cell-specific transcriptional network.^7^ Correspondingly, POU2AF2 has been shown to interact with POU2F3, a master transcriptional regulator of tuft cells,^8^ in small cell lung cancer, characterised by high *POU2F3* expression (SCLC-P).^9 10^ These studies suggest that tuft cell function may be influenced by genetic variation at 11q23.1; however, germline genetic regulation of these genes, and their relevance to human colonic epithelium and CRC risk, remain to be determined.

In this study, we nominate a key relationship between tuft cell abundance and CRC risk. We interrogate genetic regulation of 11q23.1 eQTL targets in humans by analysing genome-wide bulk and single-cell data, assessing tuft cell abundance in human colon, and experimentally delineate the function and tumourigenic potential of 11q23.1 cis-eQTL targets using genetically engineered mouse models.

## Results

### rs3087967 is the lead variant associated with 11q23.1 gene expression and CRC risk

**Figure 1:**
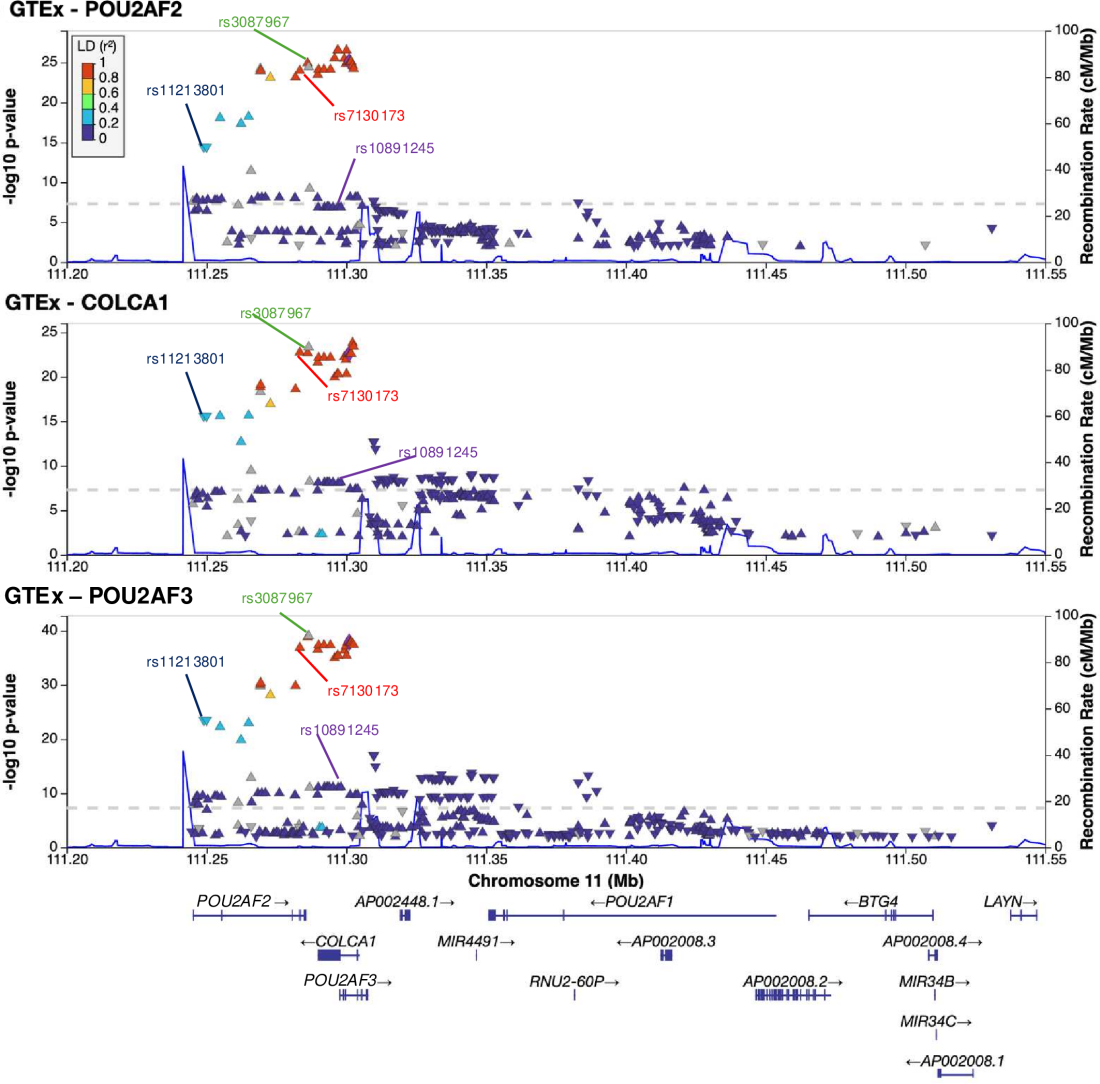
Several 11q23.1 variants exhibit significant associations with *POU2AF2, COLCA1* and *POU2AF3* expression in GTEx transverse colon RNA sequencing. LocusZoom^36^ plots of variant position and significance of association with *POU2AF2, COLCA1* and *POU2AF3* expression. Recombination rate (blue line) is derived from HapMap reference population. Variants are coloured according to their linkage disequilibrium (LD) with the tag variant (rs3802842, represented by a purple triangle), and are shown in grey if no LD information is available. Previously suggested top-associated variants are highlighted.^4 5^

Tagging SNPs are rarely found to be the causal variants in post-GWAS study, and other 11q23.1 variants demonstrate stronger association with cis-eQTL target expression than rs3802842^4 5^. To improve estimation of the relative effect of potential cis-regulatory variants at 11q23.1, we performed linear regression of expression of *POU2AF2*, *COLCA1* and *POU2AF3* on common 11q23.1 variants in GTEx transverse colon (n=367 (GTEx sigmoid colon contains only muscularis, figure 1 and online supplemental material 1). Several variants exhibited cis-eQTL effects exceeding the standard threshold of p<5e-8, including rs11213801, rs7130173 and rs3087967, with the latter conferring greatest estimated effect size among significant variants (p<5e-8) for both *COLCA1* and *POU2AF3* expression, and second-highest for *POU2AF2* (online supplemental material 1).

To investigate whether CRC risk could be delineated across eQTL effects on these genes, we tested the shared association of 11q23.1 variants with cis-eQTL target expression and CRC risk by colocalisation analysis,^11^ using summary statistics from our recent GWAS^2^ (online supplemental table S1). The expression of *POU2AF2*, *COLCA1* and *POU2AF3* was found to colocalise with CRC risk, with association at a single variant the most likely outcome in each case (Bayesian posterior probability (PPH4)=0.91, 0.99 and 1, respectively). Notably, rs3087967 was the only variant common to the 95% credible sets of all three genes, with high significance for shared genetic association at a single variant for *POU2AF2* and *POU2AF3* (PPH4=0.75 and 0.99, respectively), and a modest, but still the most likely hypothesis for *COLCA1* (PPH4=0.43) (online supplemental table S2 and online supplemental material 2). To test for independent variants with cis-eQTL effects, we repeated this analysis conditioning on rs3087967 and find no variant to be significantly independently associated with the expression of any 11q23.1 cis-eQTL target (conditional p<5e-8) (online supplemental table S3). Hence, 11q23.1 genetic variation is likely to represent a single cis-eQTL effect for *POU2AF2*, *COLCA1* and *POU2AF3* expression, with rs3087967 being the most predictive variant for both eQTL and CRC risk effects.

### 11q23.1 variation does not exhibit transcript-specific eQTL effects

Recent work highlighted a protein-protein interaction between *POU2AF2* and *POU2F3*^9 10^ and a dependence of murine small intestinal (SI) tuft cell abundance on expression of a specific *Pou2af2* transcript that encodes a POU2F interaction domain (POU2F-ID).^12^ Because of the homology between *POU2AF2*, *POU2AF3* and known POU2F interactor, *POU2AF1*, we analysed the domain composition of human *POU2AF2* and *POU2AF3* transcripts (online supplemental figure S1). Of the two annotated *POU2AF2* transcripts, only ENST00000280325 encodes the POU2F-ID. Two of six *POU2AF3* transcripts encode the POU2F-ID; ENST00000610738 and ENST00000638573 (online supplemental figure S1, orange highlight). Interestingly, the only *POU2AF2* transcript associated with CRC risk in a recent transcript isoform wide association study was the POU2F-ID transcript, potentially implicating this domain in governing 11q23.1 variation-associated CRC risk.^2^

We then assessed transcript-specific eQTL effects of 11q23.1 variants in the colon. The POU2F-ID transcript of *POU2AF2*, two non-POU2F-ID transcripts of *POU2AF3* and one POU2F-ID transcript of *POU2AF3* were strongly associated with rs3087967 genotype (p=6.7e-12, p=1.5e-11, p=8.3e-38 and p=6.8e-7, respectively). However, there is a trend of reduced expression for the non-POU2F-ID *POU2AF2* transcript. By contrast, rs3087967 and other local variants were associated with both the POU2F-ID and non-POU2F-ID transcripts of *POU2AF3* (p<5e-8), suggesting that CRC risk-associated variation at 11q23.1 is not associated with perturbation of POU2F-ID transcripts of *POU2AF2* and *POU2AF3* specifically. The functional roles of specific isoforms may still be substantial but not discernible here. Specifically, non-genetic factors such as altered transcript stability and post-translational modifications may permit such differences.

### The trans-eQTL hub at 11q23.1 influences expression of 21 distant genes, each of which confers CRC risk

To better understand 11q23.1 trans-eQTL associations, we identified trans-eQTL targets of rs3087967 across three independent datasets: (i) full thickness colonic normal mucosa samples from GTEx transverse colon RNA sequencing (RNAseq) (n=367) (online supplemental table S4), (ii) in-house stripped normal colorectal mucosa samples (n=223, SOCCS)^2^ (online supplemental table S5) and (iii) rectum normal mucosa RNAseq (n=109, INTERMPHEN)^2 13^ (online supplemental table S6). This analysis replicated associations with several targets previously identified (false discovery rate (FDR) <0.05), including: *TRPM5; SH2D6; SH2D7; HTR3E; LRMP; GNG13; MATK; OGDHL; BMX; PLCG2* and *POU2F3*, highlighting a strong overlap in the findings of these datasets and further indicating that tuft cell regulation is influenced by genetic variation at 11q23.1.

**Figure 2:**
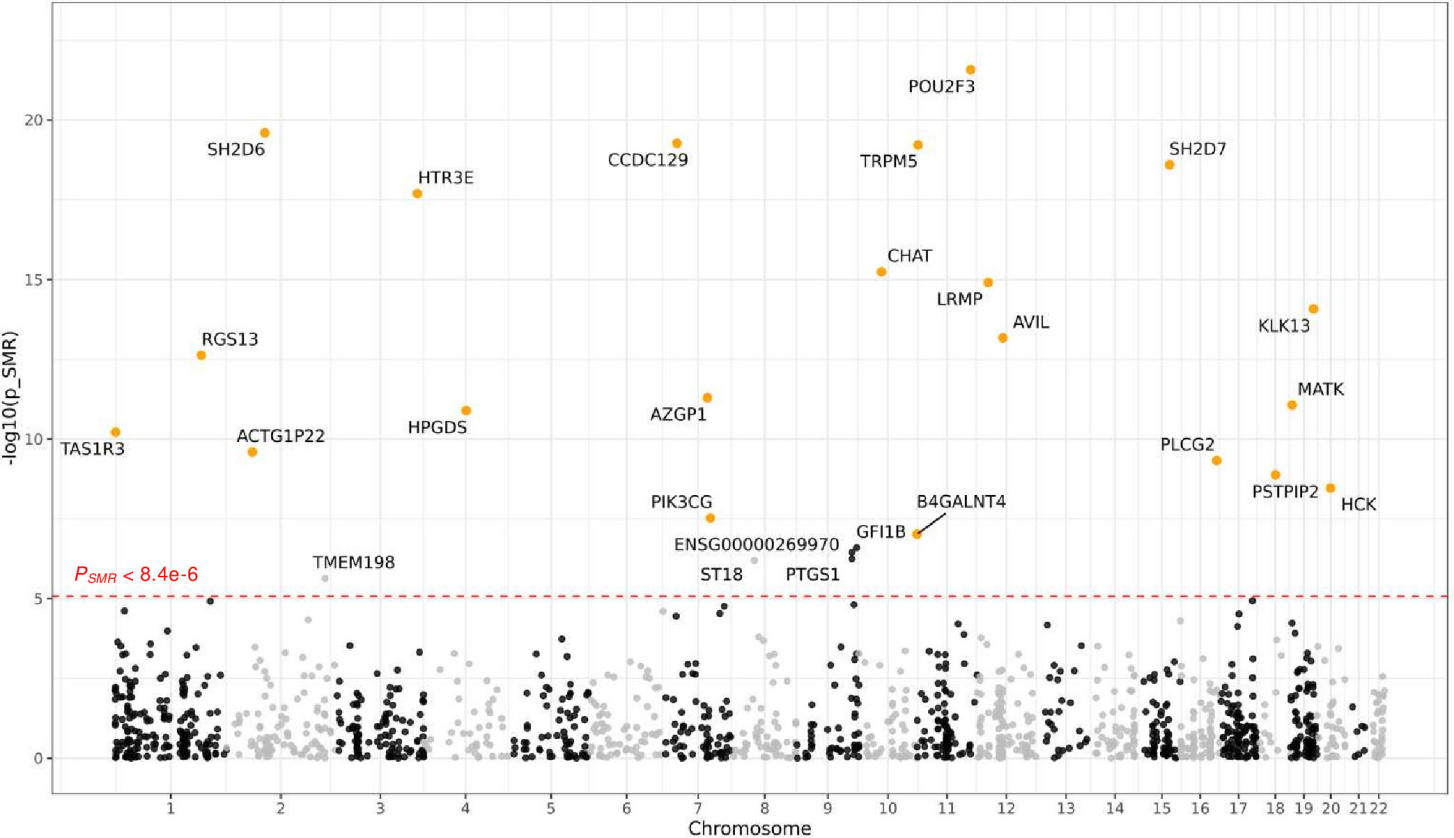
Summarised Mendelian randomisation (SMR) of 11q23.1 trans-expression quantitative trait locus (eQTL) targets identifies 21 novel genes with colorectal cancer (CRC) risk association. SMR analysis shows an association between the expression of nominally significant 11q23.1 trans-eQTL targets (p<0.01) with greater 11q23.1 trans-eQTL effect than cis-eQTL effect (n=1474) and CRC risk.^2^ 11q23.1 trans-eQTL targets with genome-wide significance for CRC risk (pSMR<8.4e-6) are highlighted and orange for those with significant trans-eQTL effects (p<5e-8).

To determine whether dysregulation of 11q23.1 trans-eQTL target genes impart CRC risk, we performed summarised Mendelian randomisation (SMR),^14^ instrumentalising genotype to infer risk conferred by trans-effects that exceeded significance of cis-effects (GTEx and SOCCS meta-analysis, nominal p<0.01, n=1474, figure 2 and online supplemental material 3). We found striking associations between decreased expression of 21 trans-eQTL target genes of rs3087967 at 11q23.1 (p<5e-8) and CRC risk, reaching genome-wide significance (SMR p<8.4e-6). These include *ACTG1P22; AVIL; AZGP1; B4GALNT4; CCDC129; CHAT; HCK; HPGDS; HTR3E; KLK13; LRMP; MATK; PIK3CG; PLCG2; POU2F3; PSTPIP2; RGS13; SH2D6; SH2D7; TAS1R3* and *TRPM5*, implicating these genes as novel CRC risk-associated loci—henceforth referred to as the ‘refined trans-eQTL targets’.

**Figure 3:**
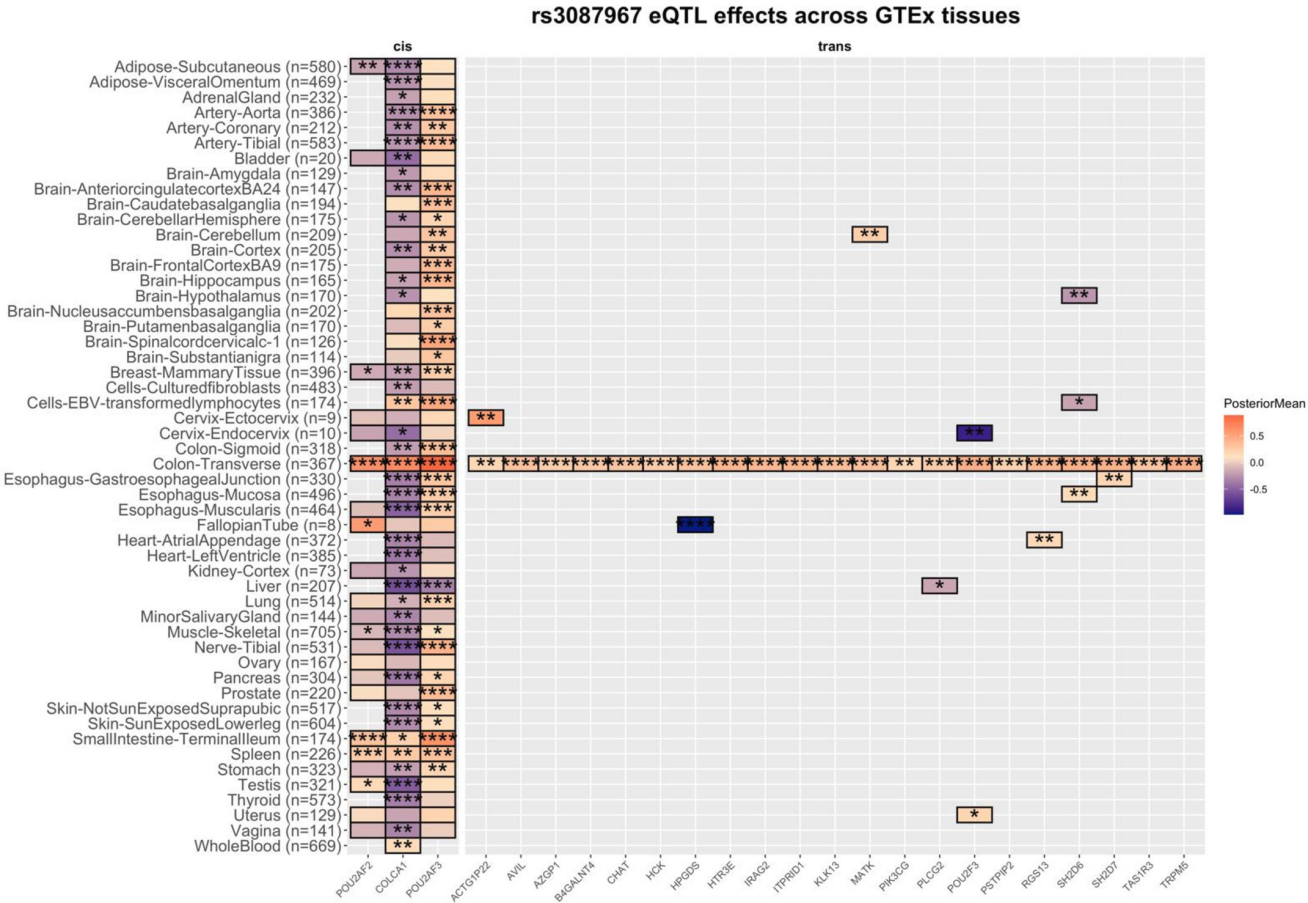
Pan-tissue 11q23.1 expression quantitative trait locus (eQTL) analysis indicates risk-associated transcriptional dynamics are largely colon-specific. Posterior mean and local false sign rate (LFSR) significance of eQTL effects of rs3087967 on 11q23.1 cis-eQTL and trans-eQTL targets across all GTEx tissue sites, calculated by multiple adaptive shrinking analysis.^15^ *P<0.05; **p<0.01; ***p<0.001; ****p<0.0001. Grey boxes indicate the absence of posterior mean statistics for tissues where the gene did not meet the minimum expression threshold (see ‘Materials and methods’ section).

Regulation of heritable disease-associated genes is often tissue specific, so as an orthogonal method to infer the causality of 11q23.1 eQTL effects we replicated our analysis across all GTEx tissue sites (n=52) and performed multiple adaptive shrinkage analysis^15^ (figure 3 and online supplemental figure S2). Compared with the transverse colon, the effect of rs3087967 on *POU2AF3* expression showed little specificity, with a shared effect direction identified in 20 tissues (local false sign rate (LFSR) <0.05), and opposite direction only in the liver. In contrast, rs3087967 effects on *COLCA1* expression occur in the opposite direction in 34 tissues, but concordant in the spleen, small intestine (terminal ileum) and whole blood. *POU2AF2*, however was tested in far fewer tissues, owing to its lower expression, but showed opposite and concordant effects in three and four tissues, respectively (figure 3), with preserved effects in the fallopian tube, testes, spleen and small intestine (terminal ileum). Remarkably, the trans-eQTL effects of rs3087967 were exclusive to the transverse colon for 13 of the trans-eQTL targets, with shared effects in the same direction (mean=0.52). The greatest magnitude of these effects was observed in the transverse colon for all targets, except for *ACTG1P22*. Together, despite their proximity, this shows 11q23.1 cis-eQTL effects may be diverse across tissues, with *POU2AF2* and *COLCA1* showing the greatest concordance with the trans-eQTL effects that are dramatically enriched in large intestine.

None of the refined trans-eQTL targets have been implicated in CRC risk by GWAS/transcriptome-wide association study (TWAS),^2^ which may be due to interaction effects with 11q23.1 genotype. To address this, we performed an interaction, case-control analysis between cis-eQTLs within 1 Mb of the refined trans-eQTL targets (Bonferroni-corrected p<0.01) and rs3087967 genotype in a meta-analysis of Generation Scotland (n=14 205 (4335 cases, 9870 controls)), the Lothian Birth Cohort (n=2550 (1032 cases, 1518 controls)) and the National Study of Colorectal Cancer Genetics (n=13 801 (6596 cases, 7205 controls)). No variant within 1 Mb of these genes was strongly associated with CRC risk based on interaction with rs3087967 genotype (minimum p=5.7e-4). We also performed a case-only interaction test, finding no evidence for risk variants surrounding the trans-eQTL targets (minimum p=0.045). Together, this indicates epistatic effects of rs3087967 do not underpin the lack of CRC risk detected at these loci by GWAS.

### POU2AF2 binding likely mediates tuft cell-specific accessibility of 11q23.1 trans-eQTL targets

While our RNAseq analyses reinforce the correlated expression of 11q23.1 cis-eQTL and trans-eQTL targets, the specific cis-eQTL target(s) responsible for governing the trans effects remain unresolved. Our previous analysis of healthy human colonic epithelium single-cell RNAseq (scRNAseq) showed 11q23.1 trans-eQTL targets preferentially correlate with *POU2AF2*, rather than *POU2AF3* or *COLCA1.*^7^ However, this analysis did not include the entire set of refined trans-eQTL targets. Using this analysis, we observed mean expression of the refined trans-eQTL targets to be overwhelmingly greatest within tuft cells, including *POU2F3* (online supplemental figure S3). Eleven of the 17 refined trans-eQTL targets that passed gene filtration were identified as tuft cell markers (FDR <0.05)^7^ (online supplemental table S7), and these preferentially correlate with one another and *POU2AF2*, in a tuft cell-specific manner (online supplemental figure S3). Addressing the exclusivity of 11q23.1 eQTL effects, though, would require single cell eQTL mapping across cell clusters of a large colorectal scRNAseq cohort with paired germline genotypes. However, such a dataset is not yet publicly available, limiting our current analysis . Meanwhile, the present analysis reinforces specificity of expression of CRC-associated 11q23.1 trans-eQTL targets, and potential shared regulation with *POU2AF2*.

**Figure 4:**
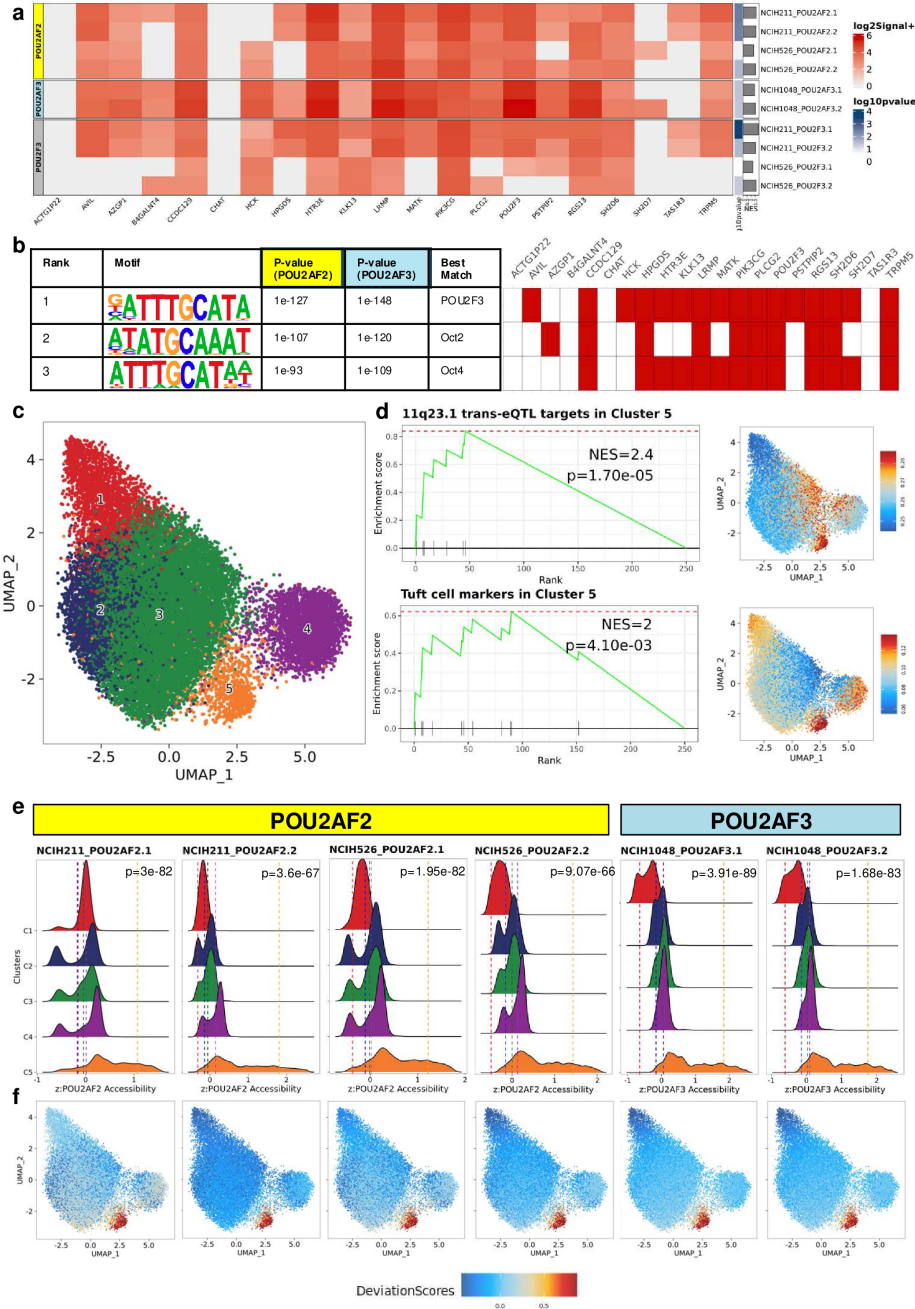
POU2AF2 and POU2AF3 are potential regulators of the colonic tuft cell chromatin accessibility landscape in the human colon. (**a**) Heatmap of signal values (log_2_+1) for POU2AF2, POU2AF3 and POU2F3 binding at refined trans-eQTL targets across NCIH211, NCIH526 and NCIH1048 SCLC-P cell lines. Gene set enrichment analysis of refined trans-eQTL targets, based on all bound sequences ranked by signal value, is also shown. (**b**) HOMER known motif enrichment results at POU2AF2-bound and POU2AF3-bound sequences surrounding refined 11q23.1 trans-eQTL targets (left). Heatmap denoting presence of core motif in sequences at refined trans-eQTL targets (right, red if present). (**c**) Uniform Manifold Approximation Projection (UMAP) embedding of single-cell ATAC sequencing (scATACseq) data from 21 620 healthy colonic epithelial cells.^17^ Colour denotes cell-cluster. (**d**) Enrichment of refined 11q23.1 trans-eQTL targets and putative colonic tuft cell signature previously defined^39^ in cluster 5 accessibility marker genes and all cells. (**e**) Distribution of z-scored accessibility of POU2AF2-bound and POU2AF3-bound sequences per-cell (denoted as ‘zPOU2AF2 Accessibility’ and ‘zPOU2F3 Accessibility’, respectively) for each antibody/cell line replicate across scATACseq clusters. Vertical line indicates the mean normalised accessibility for each cluster. P values calculated by t-test of normalised enrichment scores in cluster 5 compared with all other clusters combined. (**f**) Per-cell enrichment scores shown in (**e**) displayed on the UMAP embedding.

Given the recently proposed transcriptional activator functions of POU2AF2 and POU2AF3,^9 10^ we evaluated the binding of these proteins at refined 11q23.1 trans-eQTL targets as the potential mechanism for expression regulation. We obtained publicly available chromatin immunoprecipitation sequencing (ChIPseq) data for POU2AF2, POU2AF3 and POU2F3, along with activating transcription markers across SCLC-P cell lines (NCIH211, NCIH526 and NCIH1048), as CRC cell lines lack strong expression of 11q23.1 genes.^9^ POU2AF2 and POU2AF3 binding was assessed independently, as contributing studies indicate the expression and dependence of SCLC-P cell-line survival on these genes to be mutually exclusive.^9 16^ Occupancy of both POU2AF2 and POU2AF3 was detected at up to 17 distinct regions near 19 of the 21 refined trans-eQTL target loci (q<0.05). Non-bound targets include *ACTG1P22* and *CHAT* for both proteins, and *TAS1R3* for POU2AF3 (online supplemental figure S4a). Binding at *SH2D6* was observed at only a single surrounding region for a single POU2AF2 antibody, and only in the NCIH526 cell line. Interestingly, enrichment values were stronger for POU2AF3 binding at most genes, however this may be due to variation in the pulldown method used by the authors for each protein. Binding signatures were also enriched for the majority of refined trans-eQTL targets (p<0.05) across POU2AF2 and POU2AF3 (all replicates) and POU2F3 ChIPseq (NCIH211 only), indicating preferential occupancy of these genes (figure 4a). Sequence analysis of regions bound by POU2AF2 and POU2AF3 at refined trans-eQTL targets also showed a striking enrichment of POU2F3 binding motif (p=1e-127 and p=1e-148, respectively), detectable at 16 of these genes (figure 4b). Interrogation of sequence alignments at these regions highlighted a specific, correlated pattern of POU2AF2 and POU2F3 binding (online supplemental figure S4b). However, POU2F3 binding was not analysed alongside POU2AF3, which limits the ability to fully interpret their potential co-regulatory relationship. POU2AF2 and POU2AF3 binding is complemented by active enhancer binding proteins: p300, MED1 (NCIH211 cell line only) and H3K27 acetylation (NCIH211, NCIH526 and NCIH1048 cell lines) (online supplemental figure S4c). While this analysis provides valuable insights, it is limited by some inherent sparsity in the targeted proteins tested per cell line (eg, p300 and MED1 ChIPseq not performed alongside POU2AF3, and POU2AF3 ChIPseq performed in only a single cell line). Nonetheless, these findings suggest that both POU2AF2 and POU2AF3 are capable of transcriptionally activating the majority of 11q23.1 trans-eQTL gene targets, likely through genomic binding with POU2F3, in SCLC-P cell lines.

To test relevance of POU2AF2 and POU2AF3 genomic regulation in human colonic epithelial cells, we obtained single cell assay for transposase accessible sequencing (scATACseq) data from the healthy colorectum (n=21 620 cells).^17^ We identified five distinct cell clusters (figure 4c) with gene accessibility of cluster 5 being exclusively enriched for the refined 11q23.1 trans-eQTL targets (Normalised Enrichment Score (NES)=2.4, p=1.70e-05) and putative tuft cell marker gene set (NES=2, p=4.10e-03) (figure 4d). Notably, accessibility of *PLCG2, LRMP, HCK, PSTPIP2, PIK3CG, B4GALNT4, TRPM5* and *HTR3E* demarcates cluster 5 (FDR <0.05, online supplemental table S8), however several other refined trans-eQTL targets are highly cluster 5-specific, including *AVIL, AZGP1, B4GALNT4, CHAT, HPGDS, KLK13, MATK* and *SH2D7* (online supplemental figure S5 and online supplemental material 4). As master transcriptional regulators of differentiation, such as *POU2F3*, often function by modification of chromatin and transcriptional landscapes, we analysed the relative enrichment of all POU2AF2-bound and POU2AF3-bound sequences across the scATACseq cells and clusters (figure 4e, f). Both POU2AF2-bound and POU2AF3-bound regions were significantly enriched in cluster 5 (mean normalised accessibility range 1.07–1.8 and 1.75–1.84, respectively) compared with all other clusters (mean normalised accessibility range −0.49–0.15 and −0.65–0.08, maximum p value of t-test for difference=9.07e-66 and 1.68e-83, respectively), highlighting global enrichment of these sequences in cluster 5 specifically. POU2F3-bound region accessibility was also significantly enriched in cluster 5 (mean normalised accessibility range 1.27–1.82 vs −0.44–0.13, maximum p=2.16e-63) (online supplemental figure S6). Integration of ChIPseq with scATACseq data therefore supports the specificity of 11q23.1 trans-eQTL target expression in tuft cells and implicates both POU2AF2 and POU2AF3 as potential regulators of this process in human colonic tissue.

### CRC risk genotype at 11q23.1 is correlated with reduced tuft cell compartment

**Figure 5:**
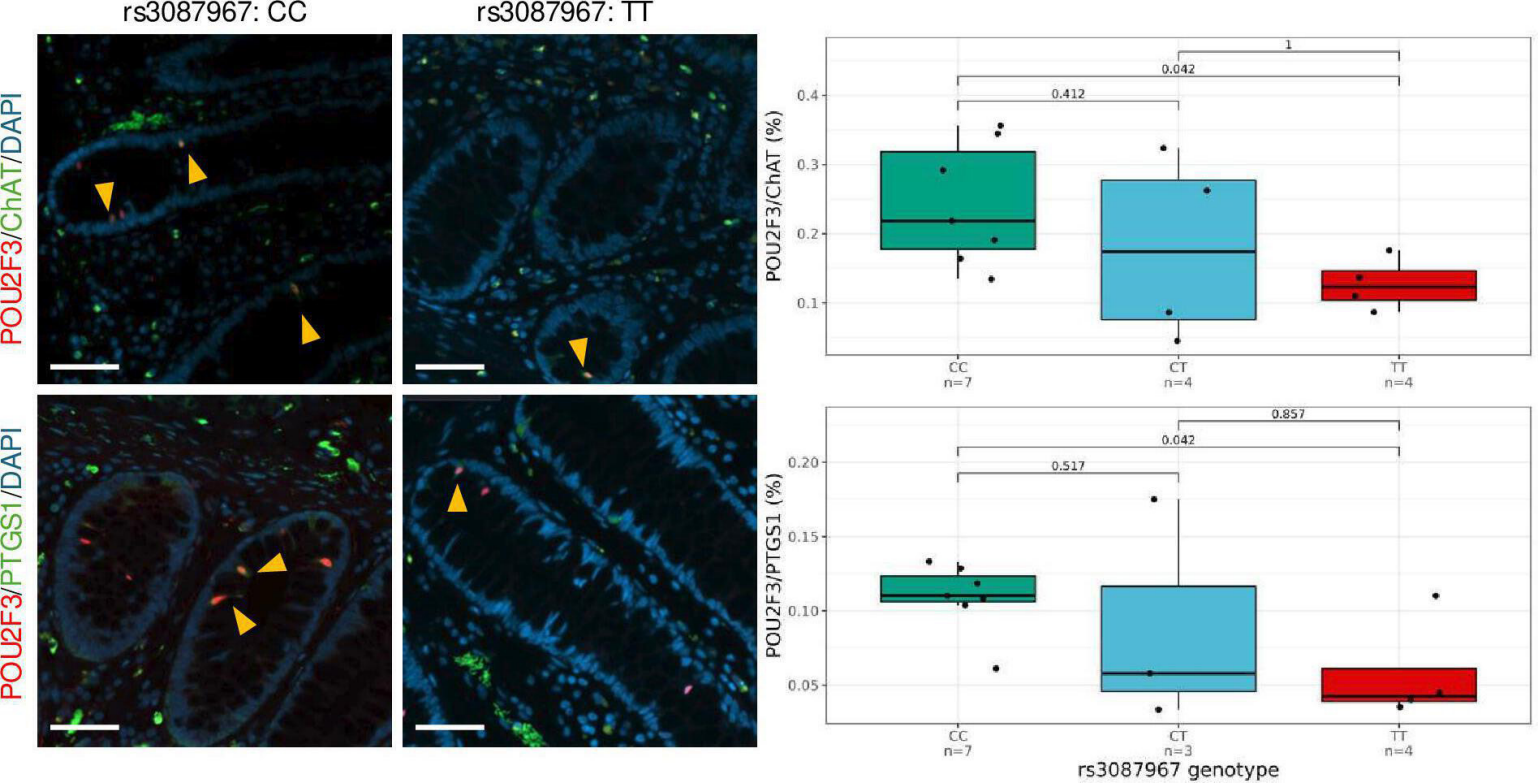
Colorectal cancer (CRC) risk genotype at 11q23.1 is associated with reduced colonic tuft cell abundance. Double immunofluorescent staining of tuft cell markers ChAT/POU2F3 (top) and PTGS1/POU2F3 (bottom) in human colon epithelium across rs3087967 genotype, C=non-CRC risk allele, T=CRC risk-associated allele. P values calculated by unpaired Wilcoxon rank-sum test. Scale bar=50 µm. Example positive cells are indicated by yellow arrowheads.

To investigate tuft cell abundance in healthy human colon across rs3087967 genotype, we developed a human colonic mucosa ‘Swiss roll’ technique to align crypts and concentrate mucosal epithelium in order to reliably detect tuft cells, estimated to represent only 2% of the colonic epithelial cell populations. Six Swiss rolls were included on a standard histological slide, each of which markedly enriched (p=4.42e-05) epithelial/stromal cell density compared with standard cross-sections: (mean=2001/mm^2^, median=1995/mm^2^, 95% CI=1709/mm^2^ to 2558/mm^2^) compared with non-rolled tissue (mean=658/mm^2^, median=482/mm^2^, 95% CI=311/mm^2^ to 1007/mm^2^) (online supplemental figure S7, onlinesupplemental videos S1  S2). We identified a significant reduction in relative abundance of cells double-positive for tuft cell markers PTGS1/POU2F3 and choline acetyltransferase ChAT/POU2F3 in individuals that were homozygous for CRC risk allele at rs3087967 (TT, n=4) compared with homozygous non-risk (CC, n=7) samples (p=0.042 for both stains) (figure 5). While we acknowledge a modest sample size, the total cell number for each stain was 1.35 million and 1.4 million cells (median of 96 846 and 81 288 cells per sample), respectively, supporting our confidence in this effect. Overall, this shows heritable variation at 11q23.1, associated with CRC risk, correlates with reduced tuft cell abundance in healthy human colon.

### Murine 11q23.1 gene knockout models replicate human transcriptional dynamics, cell abundance changes and yield diverse phenotypes

Chromatin occupancy profiling highlights *POU2AF2* and *POU2AF3* as potential regulators of the refined trans-eQTL targets in SCLC-P cell lines, with the expression these genes indicative of tuft cell abundance in the human colon. While trans-eQTL target expression is generally more specifically correlated with *POU2AF2* expression in tuft cells, the exact dependence on each of *POU2AF2* and *POU2AF3* remains to be experimentally determined. As all three 11q23.1 cis-eQTL targets are poorly expressed across CRC cell lines and highly correlated in human bulk expression, we sought to delineate the dependence of tuft cell abundance and trans-eQTL target expression on POU2AF2 and POU2AF3 in the colon by genetic perturbation of these genes in mouse models.

**Figure 6:**
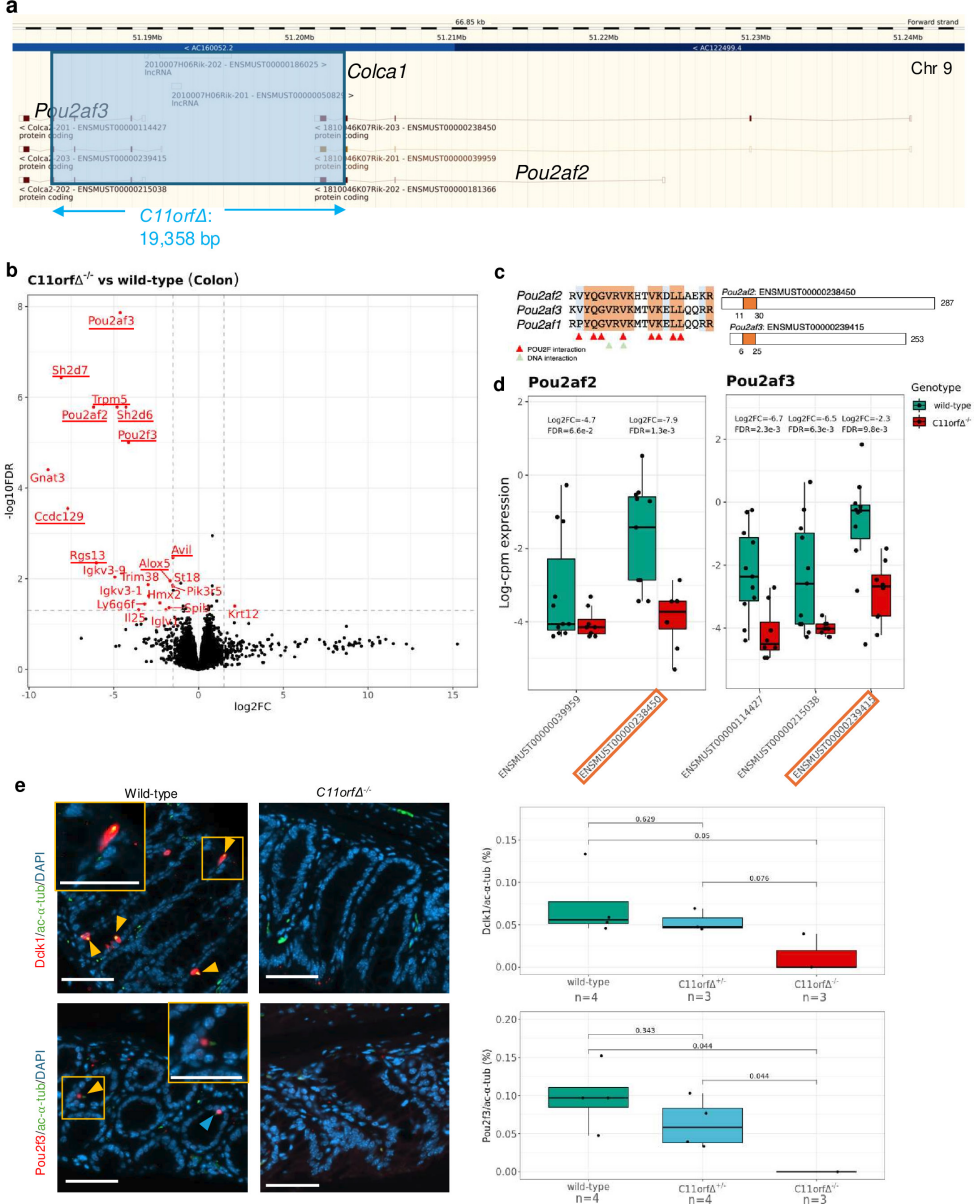
C11orfΔ^-/-^ mice recapitulate 11q23.1 variation associated transcriptional dynamics and tuft cell abundance. (**a**) Generation of the *C11orfΔ* model, characterised by a 19 358 bp deletion across all three homologs of 11q23.1 cis-expression quantitative trait loci (eQTL). Schematic obtained from ensembl (https://www.ensembl.org/, accessed 21 September 2022). (**b**) Volcano plot of the differentially expressed (DE) genes from *C11orfΔ^-/-^* (n=4) vs wild-type (n=7) colon RNA sequencing (RNAseq). Genes with absolute log_2_ fold change >1.5 and false discovery rate (FDR) <0.05 are highlighted in red and underlined if an 11q23.1 trans-eQTL target homolog. (**c**) Pou2f interaction domain homology between Pou2af1, Pou2af2 and Pou2af3 protein sequences (left) and transcripts encoding this domain (right and boxed to highlight). (**d**) Expression of detected *Pou2af2* and *Pou2af3* transcripts across *C11orfΔ^-/-^* and wild-type colon RNAseq. FDR calculated to account for the number of cis-eQTL transcripts being tested only. Orange highlight indicates transcripts encoding Pou2f interaction domain. (**e**) Immunofluorescent stains of Dclk1/acetylated-⍺-tubulin (ac-⍺-tub) and Pou2f3/ac-⍺-tub across *C11orfΔ^-/-^* genotype. Scale bar=50 μm. P values calculated by unpaired Wilcoxon rank-sum test.

To validate mice as a model of 11q23.1-associated transcriptional dynamics, we generated a knockout model of all cis-eQTL target homologs in C57/Bl6 mice via CRISPR-Cas9 (figure 6a)—known as ‘*C11orfΔ*’. Bulk RNAseq of proximal and distal colonic samples from *C11orfΔ*^−/−^ (n=4) and wild-type (n=7) mice showed reduced expression of *Pou2af2, Pou2af3*, homologs of refined 11q23.1 trans-eQTL targets: *Sh2d7, Trpm5, Sh2d6, Pou2f3, Ccdc129, Rgs13* and *Avil*, in addition to non-refined trans-eQTL target homolog, *Alox5* in *C11orfΔ*^-/-^ mice (FDR <0.05, log_2_ fold change <−1.5) (figure 6b). Unfortunately, *Colca1* expression was not assessed in this model due to poor quality alignment, but as the deleted region spans the entirety of this gene, reduced expression in *C11orfΔ*^-/-^ mice is highly likely. Altered *Pou2af2* and *Pou2af3* expression, however, was not specific to encoding of the POU2F-ID domain (figure 6c,d). Analogous to the human colon, we also observed depletion in the relative abundance of cells double positive for tuft cell markers Dclk1/ac-α-tub and Pou2f3/ac-α-tub in *C11orfΔ*^-/-^ (n=3) compared with wild-type colons (n=4, p=0.05 and p=0.044, respectively) (figure 6e). The concordant transcriptional dynamics and depletion of tuft cell abundance in association with *C11orfΔ*^-/-^ genotype validate mice as a model for this CRC risk locus, but does not delineate this effect to a specific cis-eQTL target homolog.

*C11orfΔ*^-/-^ mice also consistently weighed less than *C11orfΔ*^+/-^ and wild-type littermates and showed decreased overall survival, indicative of reduced thriving possibly linked to impaired intestinal function (online supplemental figure S8). Furthermore, male *C11orfΔ*^-/-^ mice were infertile indicating potential functions of 11q23.1 cis-eQTL targets in other tissues.

### Mouse colonic tuft cell abundance is dependent on Pou2af2, not Pou2af3 expression

**Figure 7:**
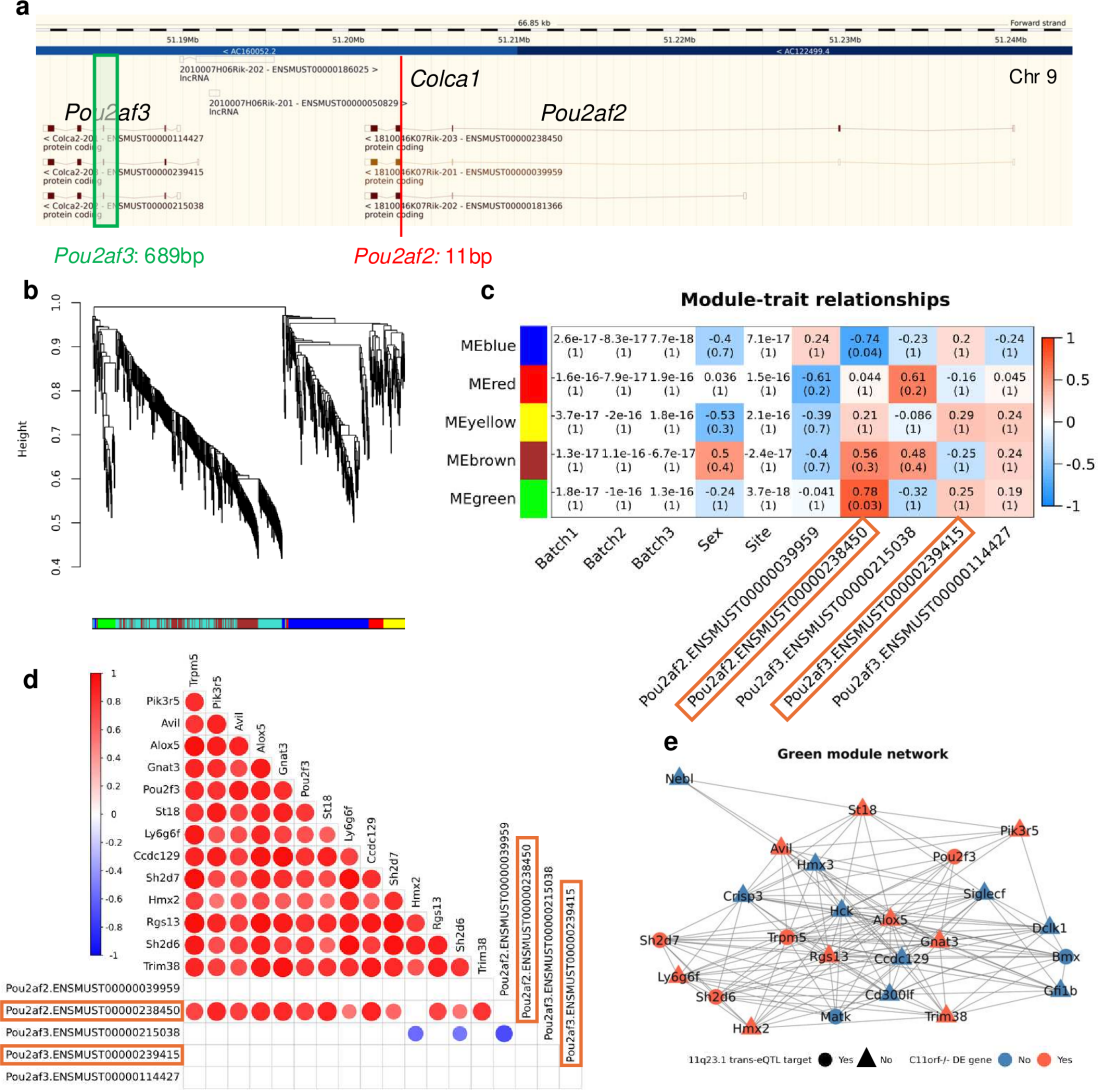
11q23.1 trans-expression quantitative trait loci (eQTL) target homolog expression is correlated with Pou2f interaction domain encoding Pou2af2 transcript specifically. (**a**) Generation of the *Pou2af2* and *Pou2af3* models. Schematic obtained from ensembl (https://www.ensembl.org/, accessed 22 September 2022). (**b**) Weighted Gene Co-expression Network Analysis of the expression of genes nominally significantly downregulated *C11orfΔ* mice (p<0.01, n=584), across *Pou2af2^-/-^* (n=2) and wild-type (n=7) colon RNA sequencing (RNAseq). (**c**) Sample trait matrix of the Pearson’s correlation (above) and false discovery rate (FDR)-corrected significance (below) between module eigengenes and sample traits, including cis-eQTL target homolog transcripts (boxed if contain POU2F-ID). (**d**) Pairwise Pearson’s correlations (p<0.05) between genes downregulated in *C11orfΔ^-/-^* mice (figure 6b) identified in the green module, in addition to *Pou2af2* and *Pou2af3* transcripts. (**e**) Kamadakawai network of green module gene relatedness (adjacency >0.3).

To interrogate the contribution of individual 11q23.1 cis-eQTL target homologs to *C11orfΔ*^-/-^ transcriptional dynamics and phenotypes, we generated an additional knockout mouse model of *Pou2af2* by CRISPR-Cas9 and obtained a *Pou2af3* model from the Canadian Mutant Mouse Repository^18^ (see ‘Materials and methods’ section and figure 7a). To identify *Pou2af2*-regulated genes, we collected RNAseq data from *Pou2af2*^-/-^ (n=2) and wild-type (n=7) colonic mucosa and subjected the expression of differentially expressed genes between *C11orfΔ*^-/-^ and wild-type mice (p<0.01, n=584) to Weighted Gene Co-expression Network Analysis (WGCNA)^19^ (figure 7b). We found five gene modules, with the eigengene (first principal component) of the green gene module being highly and significantly correlated with expression of *Pou2af2* POU2F-ID transcript (correlation=0.78, FDR=0.03), but not the *Pou2af2* non-POU2F-ID transcript (correlation=−0.041, FDR=1), or any *Pou2af3* transcripts (maximum correlation=0.25, minimum FDR=1) (figure 7c). The green module includes several genes differentially expressed between *C11orfΔ*^-/-^ and wild-type mice, including *Trpm5, Pik3r5, Avil, Alox5, Gnat3, Pou2f3, St18, Ly6g6f, Itprid1* (murine homolog of *CCDC129*), *Sh2d7*, *Hmx2, Rgs13, Sh2d6* and *Trim38*, indicating reduced expression of these genes in *C11orfΔ*^-/-^ mice to be attributable to expression of *Pou2af2* POU2F-ID transcript (online supplemental material 5). All downregulated genes in *C11orfΔ*^-/-^ mice present in this green module, excluding *Hmx2*, were correlated with expression of the *Pou2af2* POU2F-ID transcript (p<0.05, median correlation=0.76), but not the *Pou2af2* non-POU2F-ID transcript or any *Pou2af3* transcript (figure 7d). The green module hub genes (adjacency >0.3) also include several 11q23.1 trans-eQTL target homologs: *Trpm5, Pou2f3, Sh2d7, Alox5* and *Avil*, highlighting them as drivers of this correlation (figure 7e). Perturbation of *Pou2af2* expression, therefore, shows 11q23.1 trans-eQTL target homolog expression in the colon is specifically correlated with the *Pou2af2* POU2F-ID transcript, but no *Pou2af3* transcripts.

Phenotypically, *Pou2af2*^-/-^ mice weighed significantly less than both *Pou2af2*^+/-^ (p=8e-3) and wild-type mice (p=4.98e-4), which was not observed in *Pou2af3*^-/-^ mice (online supplemental figure S9). *Pou2af3*^-/-^ but not *Pou2af2*^-/-^ males were also infertile, further decoupling the function of these genes across tissues.

**Figure 8:**
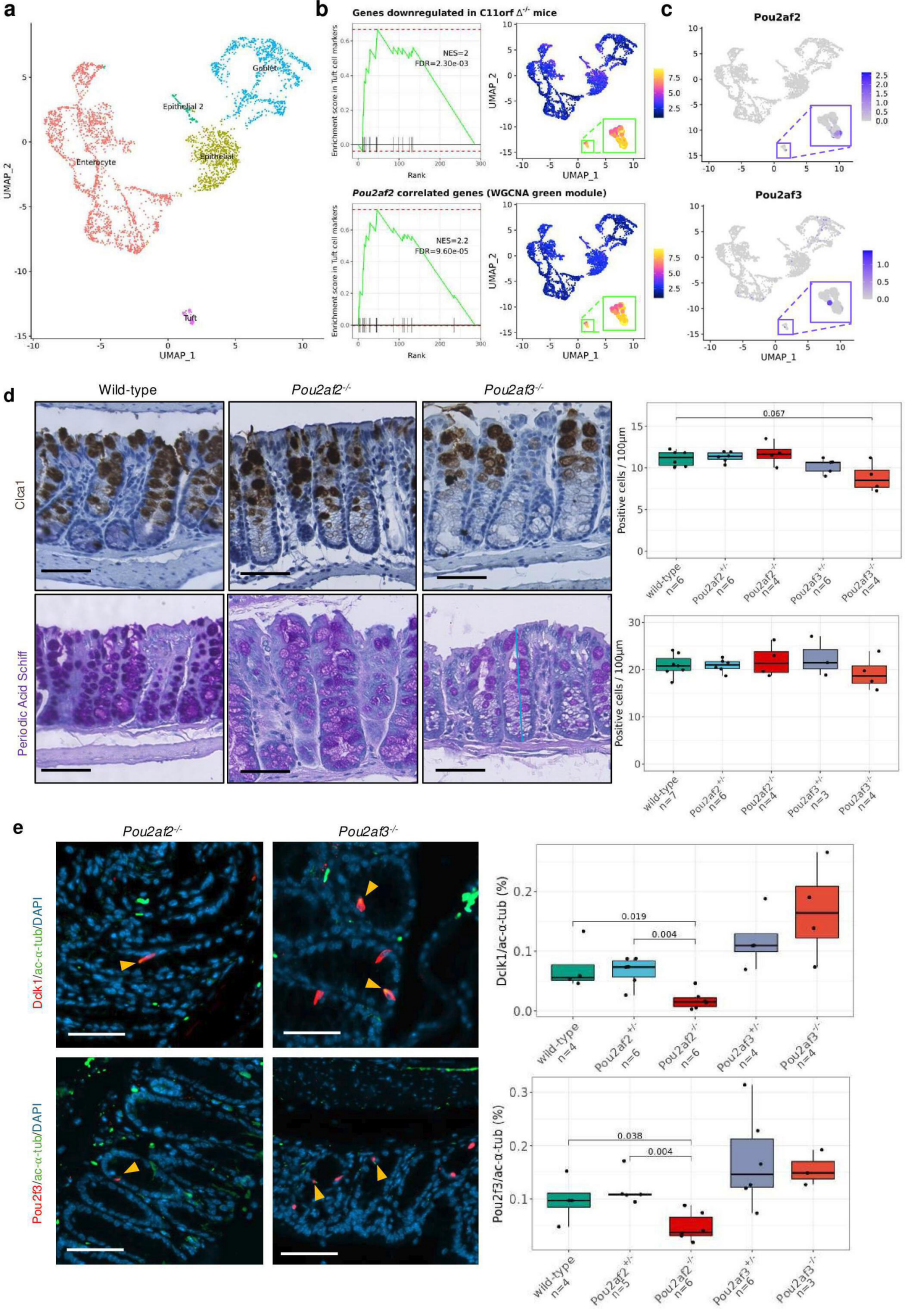
Pou2af2 and Pou2af3 expression is divergent across murine colonic epithelium and correlates with the abundance of tuft and goblet cells, respectively. (**a**) UMAP embedding of single-cell RNA sequencing (scRNAseq) from 16 828 healthy mouse colon epithelial cells.^20^ Cells are annotated by confidently annotated cell-cluster, see ‘Materials and methods’ section. (b) Gene set enrichment analysis (GSEA) (left) and single-sample GSEA (right) of *C11orfΔ^-/-^* vs wild-type differentially expressed genes (top, figure 6b) and *Pou2af2* correlated genes from the green module (bottom, figure 6c) in murine tuft cell markers and across single cells. (c) Raw expression of *Pou2af2* and *Pou2af3* across individual cells in (**a**). (d) Abundance of Clca1 and periodic acid-Schiff stains of goblet cells and mucins, respectively, across *Pou2af2* and *Pou2af3* genotypes. P values are calculated by unpaired Wilcoxon rank-sum test. Count number is normalised to the length of crypts along their axis, as shown by yellow bar. (e) Abundance of double positive cells for Dclk1/ac-⍺-tub and Pou2f3/ac-⍺-tub markers of tuft cells by immunofluoresence (figure 6e) across *Pou2af2* and *Pou2af3* genotype. P values are Benjamini-Hochberg corrections of unpaired Wilcoxon rank-sum test. Scale bar=50 μm.

We also obtained publicly available scRNAseq of wild-type mouse colonic epithelium^20^ to assess the specificity of expression of these genes across murine epithelial cells (figure 8a). Markers of murine tuft cells were significantly enriched for the genes downregulated in the *C11orfΔ*^-/-^ colon (NES=2, FDR=2.3e-03) and green module hub genes (NES=2.2, FDR=7.00e-05) (figure 8b). *Pou2af2* expression was highly specific to a subset of tuft cells, concordant with its identification as a marker of this cell-type (figure 8c and online supplemental material 6) and *Pou2af3*, while expressed in a minority of tuft cells, also shows diffuse expression across goblet cells. As there is a shared differentiation trajectory of these cell types, we analysed goblet cell abundance across *Pou2af2* and *Pou2af3* genotype by immunohistochemistry of goblet marker, Clca1, and periodic acid-Schiff (PAS) staining of neutral mucopolysaccharides, present within and secreted by goblet cells (figure 8d). Abundance of Clca1 was moderately reduced in *Pou2af3*^-/-^ (p=0.067), but not *Pou2af2*^-/-^ mice. We also observed a modest reduction of PAS staining in *Pou2af3*^-/-^ mice only. Finally, consistent with the correlation of 11q23.1 trans-eQTL targets with *Pou2af2* specifically, abundance of Dclk1/ac-⍺-tub and Pou2f3/ac-⍺-tub stained cells was significantly reduced in *Pou2af2*^-/-^ (p=0.019, p=0.038, respectively), but not *Pou2af3*^-/-^ mice (figure 8e). Taken together, molecular phenotyping of these models highlights similar 11q23.1 variation associated expression changes, and that reduced mouse colonic tuft cell abundance is a direct result of *Pou2af2* expression, but not *Pou2af3* expression.

### Pou2af2 expression is suppressive of colonic tumourigenesis

**Figure 9:**
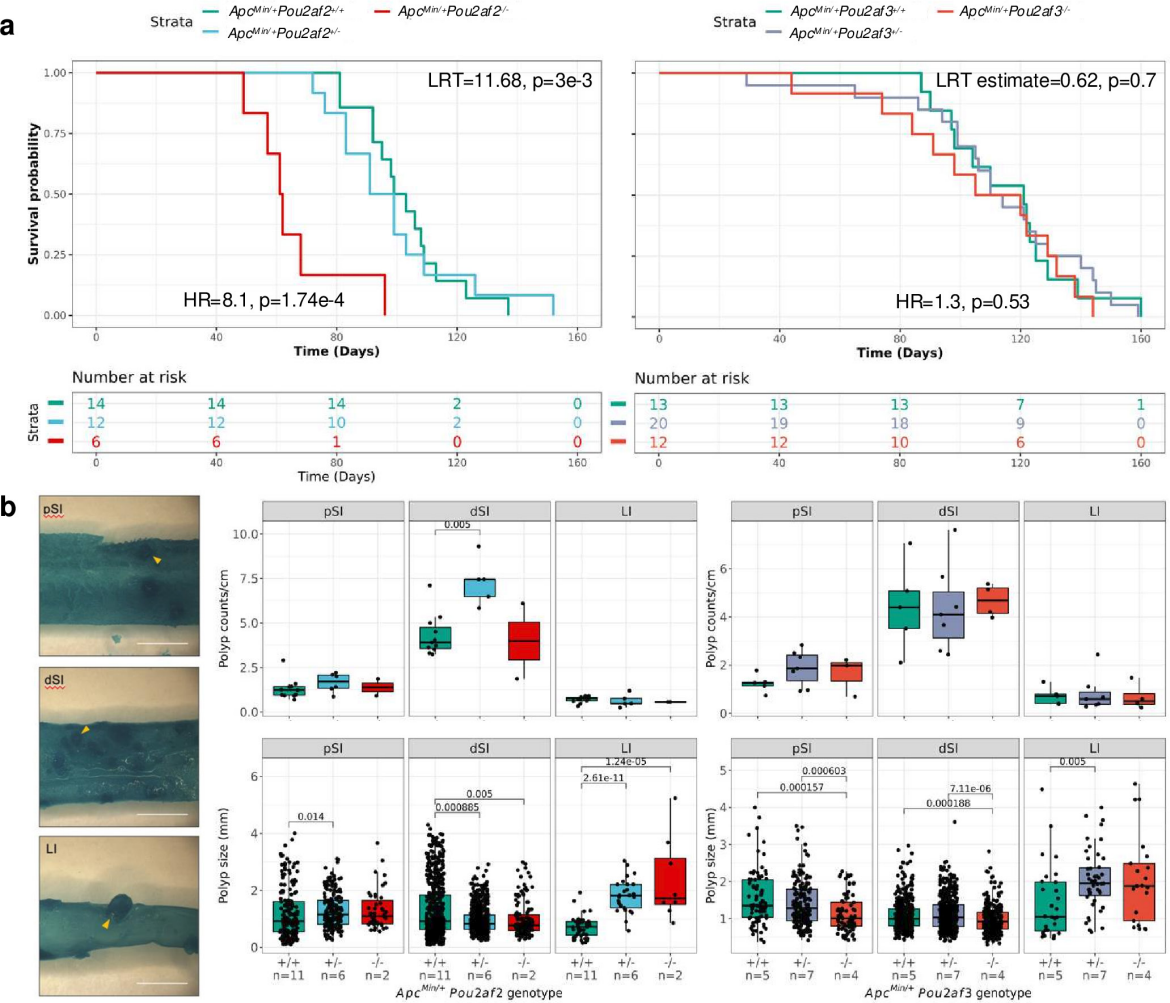
Expression of Pou2af2, but not Pou2af3, is protective of tumourigenesis in *Apc^Min/+^* mice. (**a**) Kaplan-Meier curves of *Apc^Min/+^Pou2af2* (left) and *Apc^Min/+^Pou2af3* (right) survival. Both hazard ratio (HR) of homozygous *Pou2af2* and *Pou2af3* genotypes and likelihood ratio test (LRT) statistics are shown for univariate Cox proportional hazard tests. (**b**) (Left) Example images of methylene blue stained intestinal samples, with yellow arrowhead identifying example polyps. Scale bar=1 cm. (Right) Abundance and size of methylene blue-stained polyps across *Apc^Min/+^Pou2af2* and *Apc^Min/+^Pou2af3* mice (middle and right). pSI, proximal small intestine; dSI, distal small intestine; LI, large intestine/colon. P values are Benjamini-Hochberg corrections of unpaired Wilcoxon rank-sum test.

The specific dependence of CRC risk-associated trans-eQTL target homolog expression on *Pou2af2* implies (i) 11q23.1 variation may confer CRC risk via dysregulation of this gene specifically and (ii) causal relevance of associated tuft cell abundance changes. To interrogate the tumour suppressive effects of *Pou2af2* and *Pou2af3* expression independently, we crossed single-knockout colonies onto the multiple intestinal neoplasia, *Apc^Min/+^* model and allowed mice to age until they exhibited a moribund phenotype.^21^ Interestingly, the proportion of *Apc^Min/+^Pou2af2^-/-^* mice generated was less than expected for Mendelian inheritance patterns (χ^2^ p=0.06, online supplemental table S9) potentially implicating *Pou2af2* in exacerbation of Wnt signalling regulation. *Apc^Min/+^Pou2af2^-/-^* mice showed a significant reduction in overall survival (HR=8.1, p=1.74e-4), whereas *Apc^Min/+^Pou2af3^-/-^* mice were generated at expected frequencies (p=0.19, online supplemental table S10), and showed no reduction in survival (HR=1.3, p=0.53) (figure 9a).

To investigate intestinal tumour burden, we analysed polyp frequency and size using methylene blue staining (figure 9b). Unfortunately, *Apc^Min/+^Pou2af2^-/-^* mice are under-represented, due to the difficulty in consistently generating these mice, conformation with NC3R ARRIVE guidelines^22^ and inability to collect samples due to COVID-19 restrictions. However, *Apc^Min/+^Pou2af2^+/-^* mice exhibited ~twofold increase in polyp number in distal SI compared with *Apc^Min/+^* mice (FDR=0.005), in addition to increased polyp size in proximal SI (FDR=0.014) and colon (FDR=2.61e-11). Similarly, *Apc^Min/+^Pou2af2^-/-^* mice exhibited significantly larger polyps in the colon than *Apc^Min/+^* mice (FDR=1.24e-5), further supporting *Pou2af2* to be associated with increased tumour initiation and progression on this background.

In contrast, *Pou2af3* genotype was not associated with any significant changes in tumour frequency in SI or colon of *Apc^Min/+^* mice (figure 9b). Polyps were also significantly smaller in *Apc^Min/+^Pou2af3^+/-^* and *Apc^Min/+^Pou2af3^-/-^* proximal SI (FDR=1.57e-4, FDR=6.03e-4, respectively) and distal SI (FDR=1.88e-4, FDR=7.11e-6, respectively). While there was a small increase in size of colonic polyps from *Apc^Min/+^Pou2af3^+/-^* (FDR=0.005), this was not significantly increased in *Apc^Min/+^Pou2af3^-/-^* mice.

While these findings establish a clear link between Pou2af2 expression and CRC tumourigenesis, the precise mechanistic pathways, especially concerning tuft cells, remain undetermined. Investigation into the reduction of tuft cells as a potential underlying factor should be pursued further using tuft cell-specific knockout mouse models.

## Discussion

In this study, we identify several 11q23.1 trans-eQTL targets, confirm their relevance to CRC risk, and delineate their genetic association with rs3087967. Integration of functional assay data with genome-wide, molecular characterisation of healthy human colonic epithelium at a single-cell resolution implicates both POU2AF2 and POU2AF3 as potential regulators of tuft cell-specific expression of 11q23.1 trans-eQTL targets. However, experimental investigation in single-gene knockout mice highlights *Pou2af2* to be the regulator of these effects in the colon. We also confirm CRC risk genotype at 11q23.1 to correlate with reduced tuft cell abundance in the human colon, and assess the contribution of *Pou2af2* and *Pou2af3* expression to tumourigenesis in mice. This defines a causal, oncogenic effect of *Pou2af2* depletion, and by correlation, colonic tuft cell abundance.

Convergence of CRC risk and cis-eQTL effects to rs3087967 reinforces casual relevance of cis-eQTL effects and implies this site in the underlying mechanism of gene dysregulation. Furthermore, as the trans-eQTL effects identified in the transverse colon were largely specific to this site and 11q23.1 variation associated cancer risk has not been identified in any other tissue, this suggests relevance of these transcriptional dynamics in governing CRC risk. Importantly, we statistically confirm causal relevance of 11q23.1 trans-eQTL targets by integration of eQTL effects with the largest CRC risk GWAS to date and SMR, identifying a myriad of novel CRC risk-associated genes. Diminished cis-eQTL effects at these regions support the idea that their expression is predominantly regulated by 11q23.1 trans-effects. This is consistent with their previous undetection in GWAS/TWAS and aligns with the transcriptionally activating role of POU2AF2 in colonic tissue. While the exact mechanism of cis-eQTL target dysregulation remains to be elucidated, rs3807967 lies within the 3’UTR of *POU2AF2*, suggesting this may occur by altered transcript stability and reinforcing altered *POU2AF2* as the causal gene.

Our recent work potentiated decoupling of cis-eQTL and trans-eQTL target expression,^7^ with the present study experimentally validating this. Our analysis of published ChIPseq data from lung cancer cell lines showed that both POU2AF2 and POU2AF3 are capable of regulating the expression of trans-eQTL targets. However, in the colon, we find preferential correlation of trans-eQTL target expression with *POU2AF2* in human tuft cells,^7^ segregation of their expression across the epithelium and dependence of murine colonic tuft cells on *Pou2af2* only. Variation in these findings may arise from differences in the tissue, organism and methodology, from which these datasets were derived. However, it is data derived from the colon where we find notable phenotypes that are most specific to *POU2AF2*, a finding common to both human and mouse analyses. Reassuringly, this conclusion is consistent with those of Nadjsombati *et al*,^12^ who show greater dependence of tuft cell abundance on *Pou2af2* expression in the mouse SI, and support the divergent function of these genes in colon.

Tuft cells are associated with paracrine stem-related, neurotransmitting-related and immune-related functions,^23^,^25^ including a well-characterised mechanism of defence against helminth infection.^26^ However, this is derived from the SI and cannot necessarily be extrapolated to colon. The role of tuft cell markers in governing such processes has been well documented^27^ and while not directly tested, we experimentally determine reduced tuft cell abundance to correlate with CRC risk in humans. Accordingly, reduced tuft cell abundance has been associated with pancreatic tumourigenesis in independent studies via immune-related signalling,^28 29^ implicating this as a potential route by which CRC risk occurs. Additionally, lower abundance of tuft cells has been observed in the colon of patients with quiescent UC.^30^ As there is a well-known association between colitis and CRC risk,^31^ reduced tuft cell abundance may be relevant in colitis-associated CRC (CA-CRC) also, with specific relevance of 11q23.1 genes supported by genetic mapping studies identifying the homologous region in mice to mediate susceptibility to models of CA-CRC.^32^ Notably, recent work has implicated *TET2/3* as critical mediators of the response of mice to chemically induced colitis by regulation of POU2F3-methylation and SI tuft cell abundance,^33^ further supporting the importance of this cell-type in governing immune response in gut.

While this study does not elucidate the exact mechanism by which reduced tuft cell abundance may contribute to 11q23.1 variation-associated CRC risk, accumulating functional genomic evidence highlights a novel regulator of this cell-type in the colon, *POU2AF2*, as the causal gene at this locus. This study therefore implicates a key protective role of tuft cells in human colon, indicating a potentially novel tumourigenic risk predisposing phenotype in this tissue.

## Materials and methods

### Data and code availability

Publicly available datasets used in this study and their corresponding access codes are summarised in online supplemental table S11. The GTEx V.8 data used for the analyses described in this manuscript were obtained from the GTEx Portal on 11 May 2021 and dbGaP accession number 23765. For Generation Scotland (GS) data access is through the GS access committee (access@generationscotland.org). Applications for the Lothian Birth Cohort data should be made through https://www.ed.ac.uk/lothian-birth-cohorts/data-access-collaboration. Code used to perform all analysis of this study is available at https://github.com/BradleyH017/. The graphical abstract was created using Biorender.com.

### Fine mapping and eQTL analysis

For each GTEx site analysed, the expression data were subset for those with corresponding whole genome sequence data available. Variants were subset for those which had a minor allele frequency of >0.01 within the subset data and located within 1 Mb 5’ of *POU2AF2* and 1 Mb 3’ of *POU2AF3*. As per GTEx quality control, the expression data were also subset to remove lowly expressed genes by intersection of genes that exhibited (i) >0.1 transcripts per million in at least 20% of samples, (ii) ≥6 reads in 20% of samples. Expression data were subsequently inverse normal transformed, and hidden covariates were identified using a maximum of 10 000 model iterations with PEER V.1.0^34^. Binarised sequencing batch, binarised sex and age were accounted for, in addition to a number of hidden covariates equivalent to a quarter the number of samples for each site. Residual expression was subsequently re-normally distributed and used to test association between variants and genome-wide expression using MatrixEQTL V.2.3.^35^ P value output thresholds for both local and distant associations were 0.01. The same procedure was applied to transcript-level analysis, with FDR values accounting for genome-wide multiple testing corrections. Manhattan plots were generated using LocusZoom V.0.12^36^ web interface. Comparison of gene-level rs3087967 trans-eQTL effects was performed using mashR V.0.2.79^15^ on results from the above approach across all tissues. Shared and specific effects were defined as those with an LFSR <0.05.

### Colocalisation analysis

Colocalisation analysis of gene expression and CRC risk was performed with the coloc package V.5.2.170. CRC risk GWAS summary statistics from a recent GWAS^2^ were obtained from GWAS catalogue using accession code GCST90129505. Analysis was done to assess the relative significance of global hypotheses, using a p12 parameter of 1e-4. To identify variants likely associated with the common single-variant hypothesis, a 95% credible set of variants was derived from the results.

### Conditional analysis

To perform conditional analyses, we used GCTA V.1.91.4beta^37^ software in –cojo-actual-geno mode, conditioning on rs3087967 and accounting for the distribution and frequency of variants tested in the GTEx population (for conditioning of expression) or the 1000 Genomes European reference panel (for conditioning of CRC risk).

### Summarised Mendelian randomisation

Trans-eQTL effects (>1 Mb) of 11q23.1 were calculated for whole genome sequencing variants within 1 Mb of 11q23.1 cis-eQTL targets in GTEx transverse colon (n=367) and SOCCS (n=213), as described previously (see ‘Fine mapping and eQTL analysis’ section). The summary statistics were then merged using a fixed effects model, performed using META V.1.7.^38^ Cis-eQTLs were also detected within 1 Mb of trans-eQTL target genes and meta-analysed across datasets in the same manner. SMR was then performed to assess the association between gene expression and CRC risk using SMR V.1.3.1,^14^ recently published CRC risk GWAS summary statistics^2^ and the 1000 Genomes plus UK10 000 genomes reference panel. To protect against artificial inflation of SMR significance by focussing on 11q23.1, only 11q23.1 trans-eQTLs with greater significance than any cis-eQTL detected for that gene were used in the SMR analysis.

### CRC risk interaction analysis

Genotype data were obtained for three studies, namely Generation Scotland, the Lothian Birth Cohort and the National Study of Colorectal Cancer Genetics, totalling 30 556 individuals. Details on imputation and quality control have been previously published.^13^ The interaction analysis was performed using plink V.1.90b6.18 using the epistasis function. The alleles of the lead variant at the 11q23 locus (rs3087967) were compared with all variants within 1 Mb of significant eQTLs. Significance was determined using logistic regression in the case-control analysis, and a χ^2^ test in the case only analysis. Meta-analysis of the results was performed using the R meta package.

### Transcript encoding isoform analysis

GRCh38 transcript complementary DNA (cDNA) sequences of *POU2AF2* and *POU2AF*3 were first obtained from ensembl (https://www.ensembl.org/index.html). To convert to a protein sequence, the cDNA sequence from the first AUG start codon was input into EMBOSS TransSeq (https://www.ebi.ac.uk/Tools/st/emboss_transeq/). POU2F-ID encoding was then identified, limiting search from the first amino acid to the first stop codon.

### Human scRNAseq analysis

Dimensionality reduction and clustering analysis of human colon scRNAseq data^39^ were performed as previously described.^7^ To compute correlation of gene expression at the single cell level, we extracted the expression matrix and imputed technical dropout events using Rmagic V.2.0.3.^40^ Pearson’s correlations were only computed for genes within clusters when at least 50% of comprising cells had imputed expression scores >0.01. Pearson’s correlations were only included in analysis if they passed correlation significance p<1e-3 and coefficient >0.4.

### Mouse scRNAseq analysis

Wild-type mouse colonic epithelial scRNAseq generated by Tabula Muris Consortium^20^ was obtained from gene expression omnibus using accession code GSE109774. Counts were preprocessed to remove bad quality cells as previously described.^7^ The filtered count matrix (16 828 genes and 3853 cells) was loaded into a Seurat object using Seurat V.4.1.1.^41^ Data were normalised using SCTransform before finding variable features, principal component analysis calculation and UMAP dimensionality reduction using the first 20 principal components. The kNN graph was calculated using 20 nearest neighbours and cells were clustered using a resolution of 0.6. A log transcript per 10 000 gene count matrix was also calculated as previously described.^7^

For cell annotation, a second dataset was obtained from PanglaoDB using accession number SRA653146. The authors’ cell annotations were used to calculate cluster markers based on log-normalised expression values. Gene set enrichment analysis was then performed on our analysis of Tabula Muris data, comparing it with the second dataset using fgsea V.1.16.0.^42^ Clusters significantly enriched for annotations in the second dataset (FDR <0.05) or identified through manual inspection were annotated, resulting in four confidently annotated clusters (tuft, goblet, enterocyte, epithelial) and one remaining cluster, ‘epithelial 2’. Enrichment of signatures at the single cell level was performed using escape V.1.0.0.^43^

### ChIPseq analysis and motif enrichment

NarrowPeak files of POU2AF2, POU2AF3 and POU2F3 ChIPseq results, along with BigWig files for POU2AF2, POU2F3, p300, MED1 and acetylated H3K27, were downloaded from Gene Expression Omnibus using accession code GSE18661419. The files were annotated on the hg38 genome using annotatePeaks.pl with default parameters from HOMER V.4.1.1.^44^ POU2AF2 binding was identified in each NarrowPeak file and saved as a BED file, combining data from both cell lines and antibody replicates. Motif discovery was then performed using findMotifsGenome.pl on the BED file, with the fragment size parameter set at 200 bases. To identify motif presence at each refined 11q23.1 trans-eQTL target, sequences of POU2AF2-bound regions were obtained using Biostrings V.2.60.0, and the bound sequences were searched for the motifs ATTTGCA/TTTGCAT (POU2F3), ATGCAAAT (Oct2) or ATTTGCAT (Oct4). To confirm the occupancy of POU2AF2, POU2AF3 and POU2F3 at 11q23.1 cis-eQTL and trans-eQTL targets, we summarised the maximum signal values and the number of significant peaks (q<0.05) from each experiment where these genes were the closest, using the summary statistics from the NarrowPeak files. Read enrichments of POU2F3 and POU2AF2 binding were plotted from the authors’ BigWig files using the wiggleplotr V.1.18.0 package.

### scATACseq analysis

BED files from scATACseq of healthy colonic normal mucosa^17^ were obtained from Gene Expression Omnibus using accession code GSE184462. These files were then subset for the colon epithelial samples by intersecting with cell-level metadata from this study, available on CATLAS (http://catlas.org/humanenhancer/#!/cellBrowser). All subsequent analysis was performed using the archR analysis pipeline and management package, V.1.0.1^45^: (i) an iterative LSI dimensionality reduction was generated using the tile matrix, (ii) batch correction was performed on the iterative LSI matrix across samples using Harmony V.1.081, (iii) clusters were identified using the ‘Seurat’ method and 10 nearest neighbours on the Harmony corrected matrix, (iv) UMAP embeddings were calculated on the Harmony corrected matrix, (v) accessibility around entire gene regions was calculated and used to determine cluster markers, accounting for transcription start state enrichment and log_10_(Fragment number), (vi) enrichment of POU2AF2-bound regions was performed by calculating peak annotation of the narrowpeak files for each antibody and cell-line replicate (see ChIPseq analysis and motif enrichment), (vi) mean accessibility of POU2AF2-bound regions within each cluster were manually calculated after extracting the POU2AF2 accessibility deviation matrix, (vi) p values for changes in accessibility across clusters were calculated by t-test of the accessibility in cluster 5 compared with all other clusters combined.

### Human colonic epithelium collection and processing

Following bowel resection (patient meta-data—online supplemental table S12), epithelium was removed from the full-thickness mucosa using Metzenbaum scissors, shown in online supplemental video S1. Samples were subsequently fixed in 10% neutral buffered formalin for 24 hours at room temperature and stored in 70% ethanol at 4°C. To perform Swiss-rolling, colonic tissue strips were first placed on foil-covered polystyrene with the epithelium side facing down. One end was pinned using two 25G needles, and the entire sample was stretched flat and taut by pinning with additional 25G needles. The tissue was then rolled around a toothpick towards the initially pinned end, removing the additional needles as they were encountered. Samples were then paraffin-embedded using a single 25G needle to maintain the roll structure and sectioned at a thickness of 5 μm. An example of the Swiss-rolling method is shown in online supplemental video S2.

### Genotyping of human samples

Patient blood samples were collected prior to surgery (online supplemental table S12). DNA was extracted using the Qiagen Blood and Tissue Extraction Kit (#69504), following the manufacturer’s protocol for non-nucleated blood cells. To genotype samples at rs3087967, 1.5 μL of extracted DNA was mixed with 1.25 μL of 20 μM forward primer (TGGAAGATCTGCACCACACT), 1.25 μL of 20M reverse primer (ATGCCCTCGTCCACTAACAA), 25 μL of DreamTaq Green PCR MasterMix (ThermoScientific #K1081) and 21 μL of dH_2_O. Amplification was performed by heating to 95°C for 5 min, followed by 35 rounds of 95°C for 30 s, 55°C for 30 s and 72°C for 30 s, with a final incubation at 72°C for 5 min. The PCR product was subsequently Sanger sequenced by the IGC Technical Services facility, and the rs3087967 genotype was analysed using ApE software V.2.082.

### Genetic perturbation of mice

CRISPR guide generation: plasmid px458 was digested with the restriction endonuclease BbsI (New England Biolabs, R0539S) by adding 1 μL enzyme to 1 μg of plasmid, 0.2 μL of 100X bovine serum albumin (New England Biolabs, B9000S), 200 µL of 10X NEBuffer 2.1 (New England Biolabs, B7202S) and dH_2_O to a final volume of 20 μL. The digestion reaction was then incubated at 37°C for 70 min. Guide RNA (gRNA) sequences (online supplemental table S13) were purchased as single-stranded DNA oligonucleotides, along with their reverse complements. Oligonucleotides were annealed by combining 5 μL of each primer at 100 μM, along with 10 μL of dH_2_O, and incubating at 37°C for 30 min, 95°C for 5 min then cooling to 10°C at a rate of 0.1°C/s. One μL of diluted (1:200), annealed oligonucleotides was ligated into linearised px458 by incubation with 0.5 μL of digested plasmid, 2.5 µL of dH_2_O, 5 µL of 2X T4 ligase buffer (New England Biolabs, B0202S) and 1 μL of T4 DNA ligase (New England Biolabs, M0202S) at room temperature for 30 min. Plasmids were then transformed into competent cells by mixing with 2.5 μL of the ligation mixture, followed by incubation on ice for 30 s, at 42°C for 30 s and then 5 min on ice. 950 µL of SOC medium (Thermo Fisher Scientific, 15544034) was added to each tube and incubated on shaker in a 37°C incubator for 1 hour. Each culture was plated onto two warm L-amp plates (IGC Technical Services) and the cultures were spread evenly across the entire plate using a cell spreader. Plates were incubated at 37°C overnight and examined for colonies. Colonies were picked into 5 mL L-broth with ampicillin. Cultures were incubated on shaker in a 37°C incubator overnight. Plasmids were then extracted from the expanded colonies using the QIAprep Spin Miniprep Kit (Qiagen, 27104), according to the manufacturer’s instructions.

Injection into mice: extracted plasmids were thawed on ice. For each gRNA combination, an injection mix was prepared containing 50 ng/µL of Cas9 mRNA (Tebu Bio, L-6125-20), 25 ng/µL of each gRNA, 150 ng/µL of each repair template and dH_2_O to final volume of 50 µL. This mix was then injected into C57BL/6 embryos by the BRF/Evans Transgenic Unit.

The *Pou2af3* mouse model (alternative name: Gm684_tm1c_C08, code: ABF, Strain Name: C57BL/6N-Colca2/Tcp, MGI:7257808) was obtained from the Canadian Mutant Mouse Repository.^18^ The *Pou2af2* knockout model contains an 11 bp deletion in exon 4, resulting in a frameshift mutation that affects all Pou2af2 transcripts (figure 7a). Meanwhile the *Pou2af3* model is characterised by a 689 bp deletion spanning the entire third exon, which is common to all *Pou2af3* transcripts, resulting in a premature stop codon.

### Dissection of mouse intestinal tissue

Mice were euthanised by cervical dislocation. For dissection, animals were pinned on their backs, and an incision was made along ventral midline. Small intestine samples were collected from the duodenum to just before the caecum and were divided into proximal and distal fractions. The colon was collected from (but not including) the caecum to (and including) the rectum. Samples were washed with phosphate-buffered saline (PBS), inverted on skewers and then fixed in 10% neutral buffered formalin for 24 hours at room temperature. Following fixation, these samples were rolled along their length using a toothpick and then paraffin-embedded. For staining, slides were cut at a thickness of 5 μm. For RNA collection, 1 cm of PBS-washed tissue was collected from the proximal end of each fraction, with the colon further divided into proximal and distal segments by cutting it in half along its length from the caecum to the rectum. Samples were stored in RNAlater (Invitrogen) until extraction.

### RNA extraction and sequencing of mouse colonic mucosa

For mouse tissue, RNA was extracted using the phenol-chloroform extraction method and sequenced by polyA selection on a NextSeq machine, generating 25 million paired-end reads at the Wellcome Trust Clinical Research Facility.

### Bulk RNAseq and WGCNA analysis of mouse colonic mucosa

Raw sequencing reads were aligned to the mm10 genome using Nextflow V.21.04.2 nf-core RNAseq sequence alignment pipeline.^46^ Unfortunately, this failed to accurately align reads to the *Colca1* gene, so subsequent differential and correlation analyses do not include expression quantification from this gene. Gene and transcript level counts were subset to remove lowly expressed genes as before (see ‘Fine mapping and eQTL analysis’ section). For differential expression analysis, sex, site and batch were included in the design matrix that was used to perform differential expression by quasi-likelihood F-test with edgeR V.3.34.1.^47^

For network analysis, counts were z-scored, and batch and site effects were removed using limma V.3.48.386 *RemoveBatchEffect* function. The data were then subset to include genes differentially expressed between *C11orfΔ* and wild-type mice, and used as input for WGCNA V.1.6939. The recommended scale-free topology threshold of 10 was applied for downstream analysis.

### Immunofluorescent staining of colonic tissue

To deparaffinise the tissue, samples were incubated in xylene for 15 min, followed by sequential washes in decreasing ethanol concentrations (100%, 90% and 70%) for 10 min each. Slides were then rinsed in de-ionised water (dH_2_O), followed by a 5 min incubation in 0.1% Tween in PBS. Antigen retrieval was performed by incubating slides in 1 L of prewarmed 1X Citrate Buffer (Sigma-Aldrich #C9999) with 0.1% Tween in dH_2_O for 15 min (human) or 10 min (mouse) using a pressure cooker. Slides were then cooled at room temperature for 1 hour after adding 1 L of dH_2_O. Samples were transferred to a Sequenza slide rack (ThermoScientific #73310017) with coverplates (ThermoScientific #72110017) and washed with PBS with with Tween 20 (PBS-T) to check for adherence. Permeabilisation was performed with 500 µL of 0.5% Triton-X-100 (BioXtra #T928) for 20 min at room temperature. Slides were then washed with PBS-T once more before blocking for 1 hour at room temperature with 10% goat serum (Abcam #ab7481) for human samples, or 1% bovine serum albumin (Sigma #05482) in PBS-T for mouse samples. After blocking, 300 µL of primary antibody was added to the slides and incubated at 4°C for 24 hours. All antibodies and their concentrations used in this study are summarised in online supplemental table S14.

Following primary antibody incubation, slides were washed with PBS-T three times. Then, 300 µL of secondary antibody was added to the slides and incubated at room temperature for 2 hours. After incubation, slides were washed with PBS-T five times, followed by DAPI (4',6-diamidino-2-phenylindole) staining. Finally, slides were mounted with one drop of VectaShield Antifade mounting medium with DAPI (Vectashield, #H-1200).

To analyse immunofluorescent staining, slides were scanned at 20X magnification on a Zeiss Axioscan.Z1 using Zen Blue software V.3.389 (https://www.zeiss.com/microscopy/en/products/software/zeiss-zen.html). Only double-positive cells were deemed as positive. These cells were counted blindly by eye in a single Swiss-roll section only. Background cell numbers were calculated using the ‘Cell detection’ feature of QuPath V.0.2.387, with default parameters, after colour balance removal of the red and green signals. Muscle, fat and skin nuclei were excluded from the background cell count. For comparison of cell numbers between the Swiss-rolled and non-Swiss-rolled methods, the background epithelial/stromal cell number for non-Swiss-rolled tissue was calculated as described above. Both Swiss-rolled and non-Swiss-rolled counts were then normalised to the squared footprint area occupied by the scanned tissue.

### Immunohistochemical staining of mouse colon

Deparaffinisation was performed as described previously (see ‘Immunofluorescent staining of colonic tissue’ section). Prior to the first incubation in PBS-T, slides were incubated in 3% hydrogen peroxide in PBS for 20 min at room temperature. After incubation with the secondary antibody (online supplemental table 14), nuclei were stained by the addition of 300 µL DAB (3,3'-diaminobenzidine) solution (Vector #SK-4100), following the manufacturer’s protocol, and left at room temperature for 5 min. Slides were subsequently removed from the Sequenza slide rack and transferred to dH_2_O for 5 min. Slides were then placed in Harris haematoxylin (Sigma-Aldrich #HHS32) for 30 s, followed by a wash with tap water for 5 min. Samples were then placed in increasing concentrations of ethanol (70%, 90%, 100%) for 10 min each, and subsequently in xylene for 15 min. Finally, slide covers were mounted using Cytoseal XYL mounting medium (ThermoScientific #8312-4).

Images were acquired using a Hamamatsu NanoZoomer XR and analysed with QuPath V.0.2.3.^48^ Positive cells were manually counted in each of 40 crypts across a single entire colon Swiss roll, and the counts were normalised to the length of each crypt along its axis (base to peak).

### Periodic acid-Schiff staining of mouse intestinal tissue

Slides were deparaffinised and rehydrated as described previously (see ‘Immunofluorescent staining of colonic tissue’ section). The sections were then incubated in 1% periodic acid solution for 5 min, followed by rinsing with distilled water. The slides were then covered with PAS reagent for 20 min and rinsed under tap water for 5 min. Counterstaining was performed using Harris haemotoxylin (Sigma-Aldrich #HHS32), followed by dehydration through 70%, 90% and 100% ethanol, with 10 min incubations at each concentration. Finally, slides were incubated in xylene for 15 min before mounting (see ‘Immunohistochemical staining of mouse colon’ section).

### *Apc^Min/^* ^+^ *survival and tumour burden analysis*

*Pou2af2* and *Pou2af3* mice were crossed onto a C57Bl6 *Apc^Min/+^* strain and aged until moribund phenotypes were observed, defined by the following criteria: pale paws, rectal bleeding (most common), hunched posture, starry coat or rectal prolapse. Survival analysis was carried out using survival V.3.3.1, which calculated univariate likelihood ratio test estimates and p values. Univariate tests revealed no significant interaction between sex and survival.

Intestinal samples were stained by dipping into a 0.2% methylene blue solution (Sigma-Aldrich, #M9140) diluted five to six times in dH_₂_O. Tissues were then allowed to de-stain for at least 1 week by incubating in 70% ethanol, with the ethanol changed every 2–3 days. After de-staining, samples were imaged at 0.8× magnification using a stereomicroscope. Polyp size was measured as the diameter along the axis of the tissue sample, and the analysis was performed using ImageJ V.1.52a.^49^

## Supplementary material

10.1136/gutjnl-2024-332121 online supplemental file 1

10.1136/gutjnl-2024-332121 online supplemental file 2

10.1136/gutjnl-2024-332121 online supplemental file 3

10.1136/gutjnl-2024-332121 online supplemental file 4

10.1136/gutjnl-2024-332121 online supplemental file 5

10.1136/gutjnl-2024-332121 online supplemental file 6

10.1136/gutjnl-2024-332121 online supplemental file 7

10.1136/gutjnl-2024-332121 online supplemental file 8

10.1136/gutjnl-2024-332121 online supplemental file 9

10.1136/gutjnl-2024-332121 online supplemental file 10

10.1136/gutjnl-2024-332121 online supplemental file 11

10.1136/gutjnl-2024-332121 online supplemental file 12

10.1136/gutjnl-2024-332121 online supplemental file 13

10.1136/gutjnl-2024-332121 online supplemental file 14

10.1136/gutjnl-2024-332121 online supplemental file 15

10.1136/gutjnl-2024-332121 online supplemental file 16

10.1136/gutjnl-2024-332121 online supplemental file 17

10.1136/gutjnl-2024-332121 online supplemental file 18

## Data Availability

All data relevant to the study are included in the article or uploaded as supplementary information.
