# Supplementary material for: Genetic variation at 11q23.1 confers colorectal cancer risk by dysregulation of colonic tuft cell transcriptional activator POU2AF2
Source: Gut. 2024 Nov 28;74(5):e332121. doi: 10.1136/gutjnl-2024-332121 (PMC12013567; doi:10.1136/gutjnl-2024-332121)
Supplement: online supplemental file 1 [file gutjnl-74-5-s001.pdf]

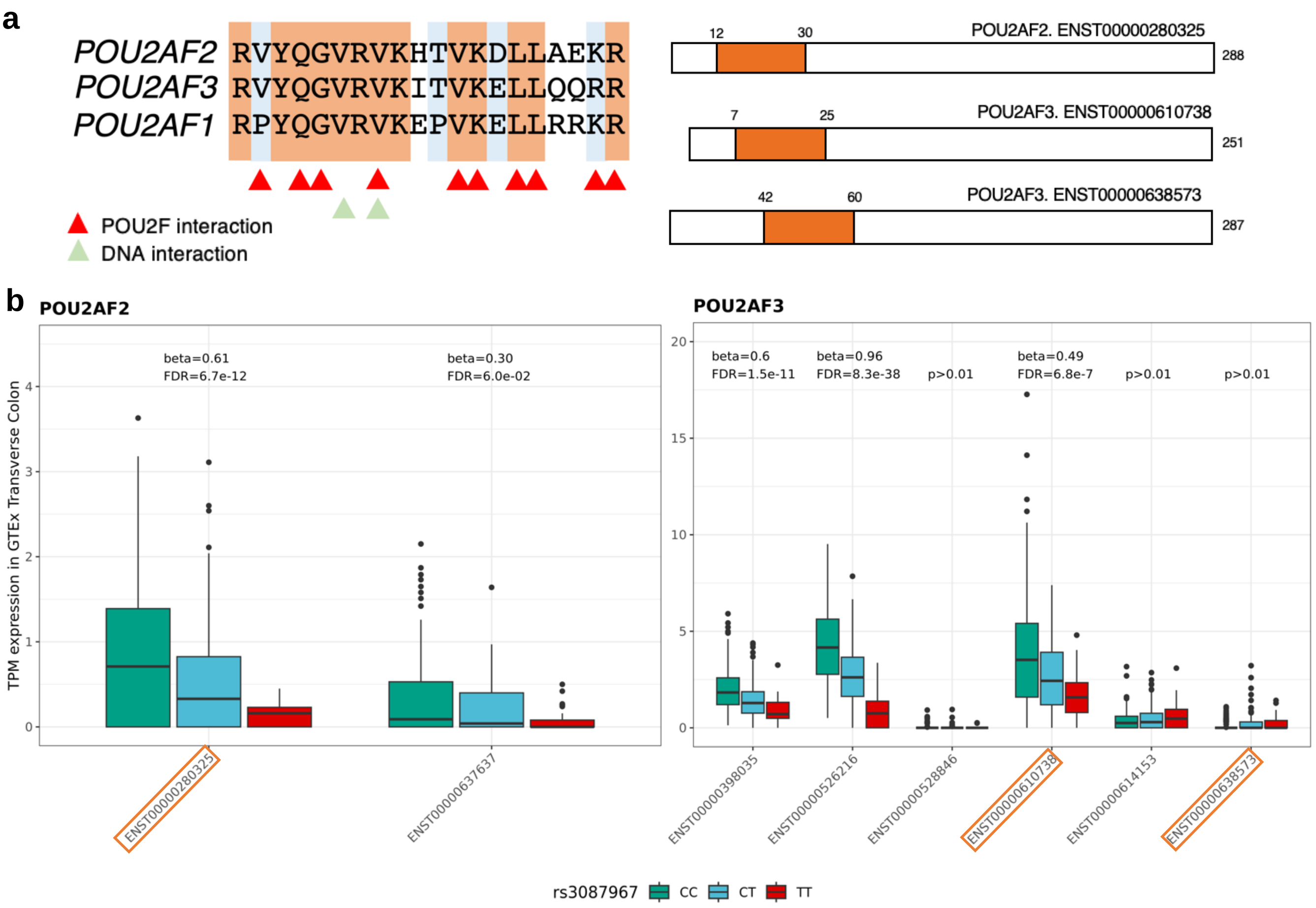

Figure S1. Transcript-specific expression and domain composition of *POU2AF2* and *POU2AF3.* (a) Sequence and encoding of POU2F interaction domains across *POU2AF2* and *POU2AF3* transcripts. Adapted from Wu *et al.,*[9]. (b) Expression of *POU2AF2* and *POU2AF3* transcripts across rs3087967 genotype in GTEx transverse colon: CC=174, CT=160, TT=33. Beta values and False discovery Rate (FDR) calculated by transcript-wide eQTL analysis. Orange highlighted transcripts encode POU2F interaction domain.
