# Supplementary material for: Genetic variation at 11q23.1 confers colorectal cancer risk by dysregulation of colonic tuft cell transcriptional activator POU2AF2
Source: Gut. 2024 Nov 28;74(5):e332121. doi: 10.1136/gutjnl-2024-332121 (PMC12013567; doi:10.1136/gutjnl-2024-332121)
Supplement: online supplemental file 2 [file gutjnl-74-5-s002.pdf]

a

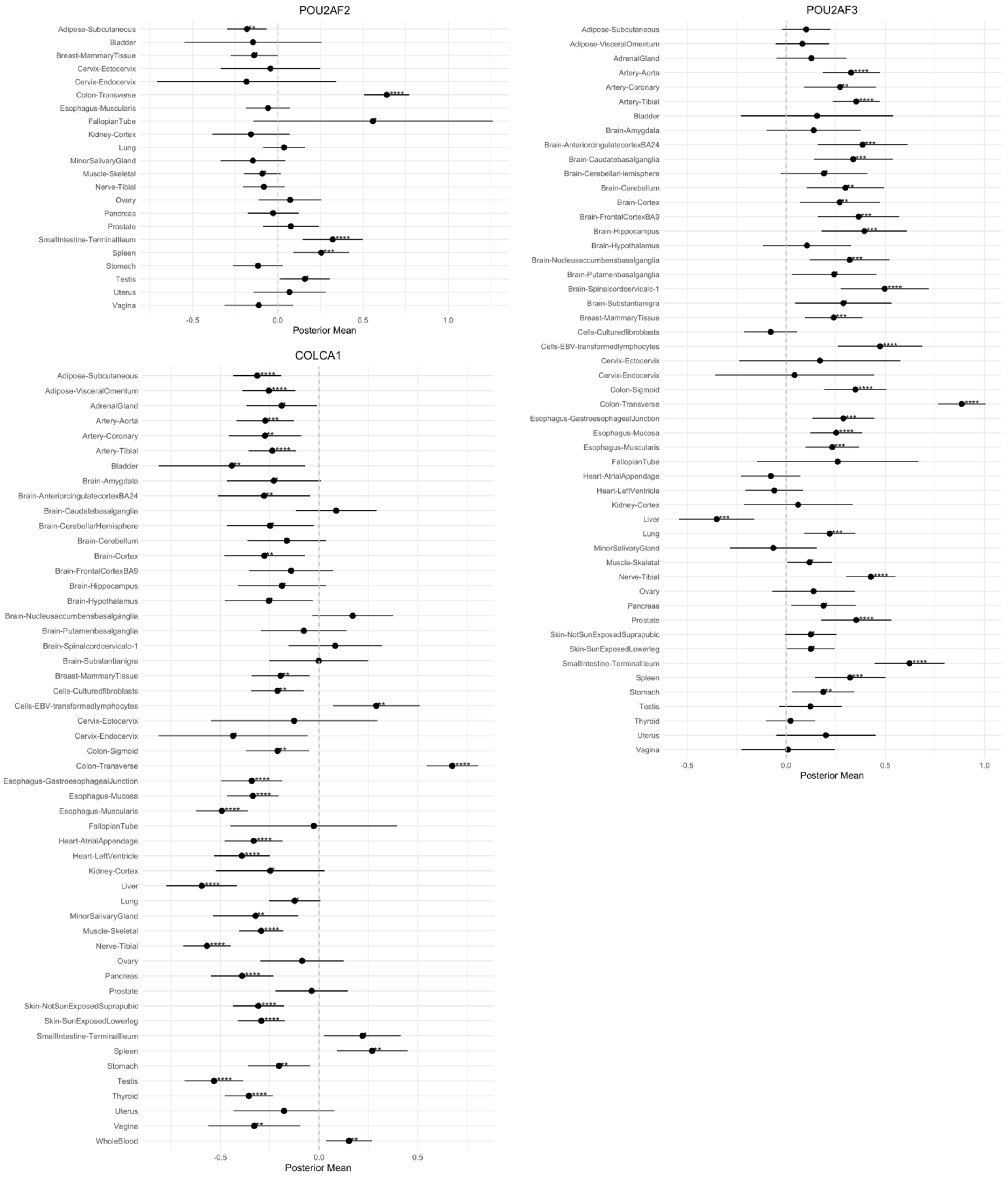
POU2AF2
Adipose-Subcutaneous
Bladder
Breast-MammaryTissue
Cervix-Ectocervix
Cervix-Endocervix
Colon-Transverse
Esophagus-Muscularis
FallopianTube
Kidney-Cortex
Lung
MinorSalivaryGland
Muscle-Skeletal
Nerve-Tibial
Ovary
Pancreas
Prostate
SmallIntestine-TerminalIleum
Spleen
Stomach
Testis
Uterus
Vagina
-0.5
0.0
0.5
1.0
Posterior Mean
COLCA1
Adipose-Subcutaneous
Adipose-VisceralOmentum
AdrenalGland
Artery-Aorta
Artery-Coronary
Artery-Tibial
Bladder
Brain-Amygdala
Brain-AnteriorcingulatecortexBA24
Brain-Caudatebasalganglia
Brain-CerebellarHemisphere
Brain-Cerebellum
Brain-Cortex
Brain-FrontalCortexBA9
Brain-Hippocampus
Brain-Hypothalamus
Brain-Nucleusaccumbensbasalganglia
Brain-Putamenbasalganglia
Brain-Spinalcordcervicalc-1
Brain-Substantianigra
Breast-MammaryTissue
Cells-Culturedfibroblasts
Cells-EBV-transformedlymphocytes
Cervix-Ectocervix
Cervix-Endocervix
Colon-Sigmoid
Colon-Transverse
Esophagus-GastroesophagealJunction
Esophagus-Mucosa
Esophagus-Muscularis
FallopianTube
Heart-AtrialAppendage
Heart-LeftVentricle
Kidney-Cortex
Liver
Lung
MinorSalivaryGland
Muscle-Skeletal
Nerve-Tibial
Ovary
Pancreas
Prostate
Skin-NotSunExposedSuprapubic
Skin-SunExposedLowerleg
SmallIntestine-TerminalIleum
Spleen
Stomach
Testis
Thyroid
Uterus
Vagina
WholeBlood
-0.5
0.0
0.5
Posterior Mean
POU2AF3
Adipose-Subcutaneous
Adipose-VisceralOmentum
AdrenalGland
Artery-Aorta
Artery-Coronary
Artery-Tibial
Bladder
Brain-Amygdala
Brain-AnteriorcingulatecortexBA24
Brain-Caudatebasalganglia
Brain-CerebellarHemisphere
Brain-Cerebellum
Brain-Cortex
Brain-FrontalCortexBA9
Brain-Hippocampus
Brain-Hypothalamus
Brain-Nucleusaccumbensbasalganglia
Brain-Putamenbasalganglia
Brain-Spinalcordcervicalc-1
Brain-Substantianigra
Breast-MammaryTissue
Cells-Culturedfibroblasts
Cells-EBV-transformedlymphocytes
Cervix-Ectocervix
Cervix-Endocervix
Colon-Sigmoid
Colon-Transverse
Esophagus-GastroesophagealJunction
Esophagus-Mucosa
Esophagus-Muscularis
FallopianTube
Heart-AtrialAppendage
Heart-LeftVentricle
Kidney-Cortex
Liver
Lung
MinorSalivaryGland
Muscle-Skeletal
Nerve-Tibial
Ovary
Pancreas
Prostate
Skin-NotSunExposedSuprapubic
Skin-SunExposedLowerleg
SmallIntestine-TerminalIleum
Spleen
Stomach
Testis
Thyroid
Uterus
Vagina
-0.5
0.0
0.5
1.0
Posterior Mean

b

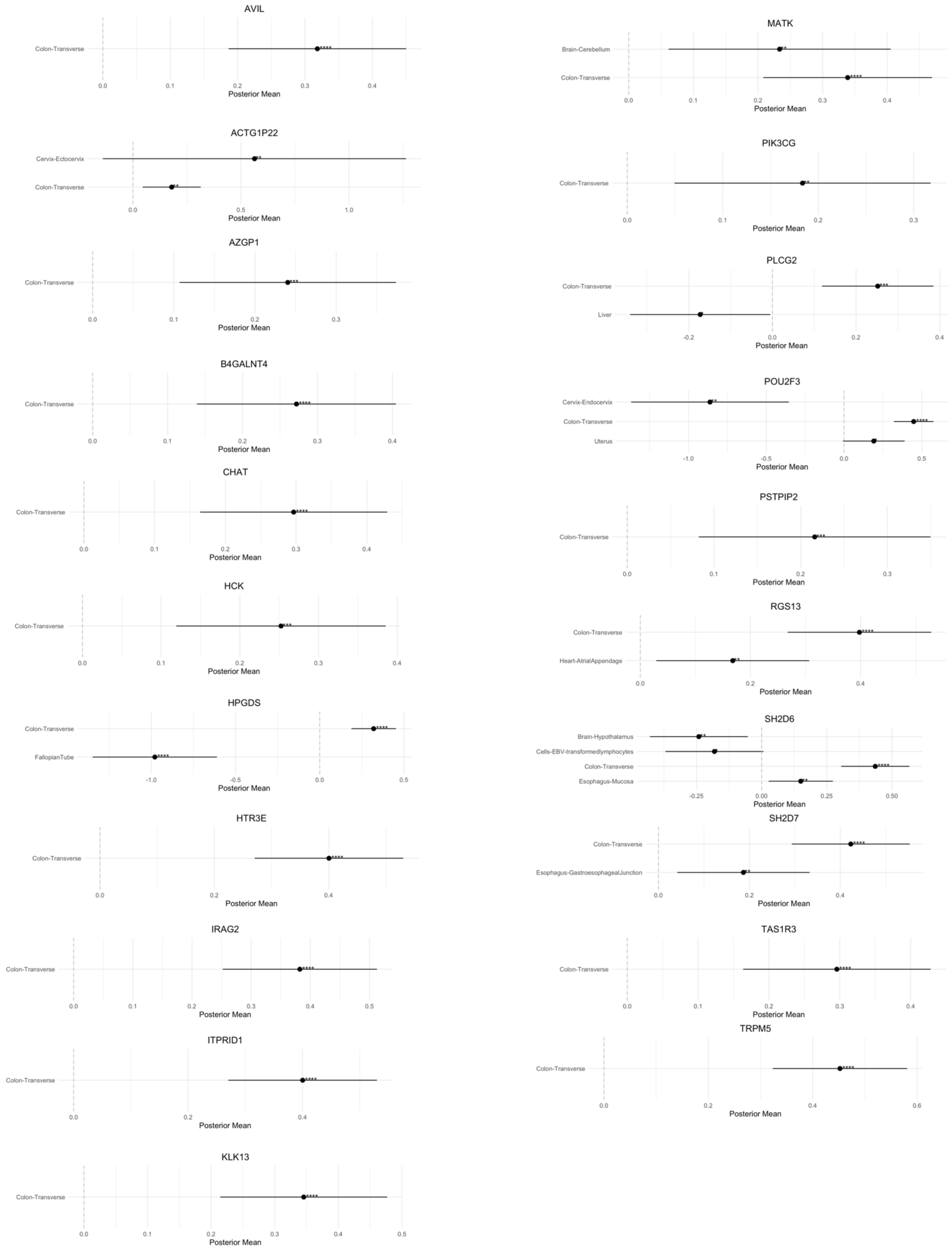

Figure S2. Differential eQTL effects of rs3087967 across GTEx tissues. Estimated effect size and standard errors of rs3087967 trans-eQTL effects across GTEx tissue sites, identified by multiple adaptive shrinking analysis[15]. Genes are only tested if they pass expression filters (see Methods).
