# Supplementary material for: Genetic variation at 11q23.1 confers colorectal cancer risk by dysregulation of colonic tuft cell transcriptional activator POU2AF2
Source: Gut. 2024 Nov 28;74(5):e332121. doi: 10.1136/gutjnl-2024-332121 (PMC12013567; doi:10.1136/gutjnl-2024-332121)
Supplement: online supplemental file 3 [file gutjnl-74-5-s003.pdf]

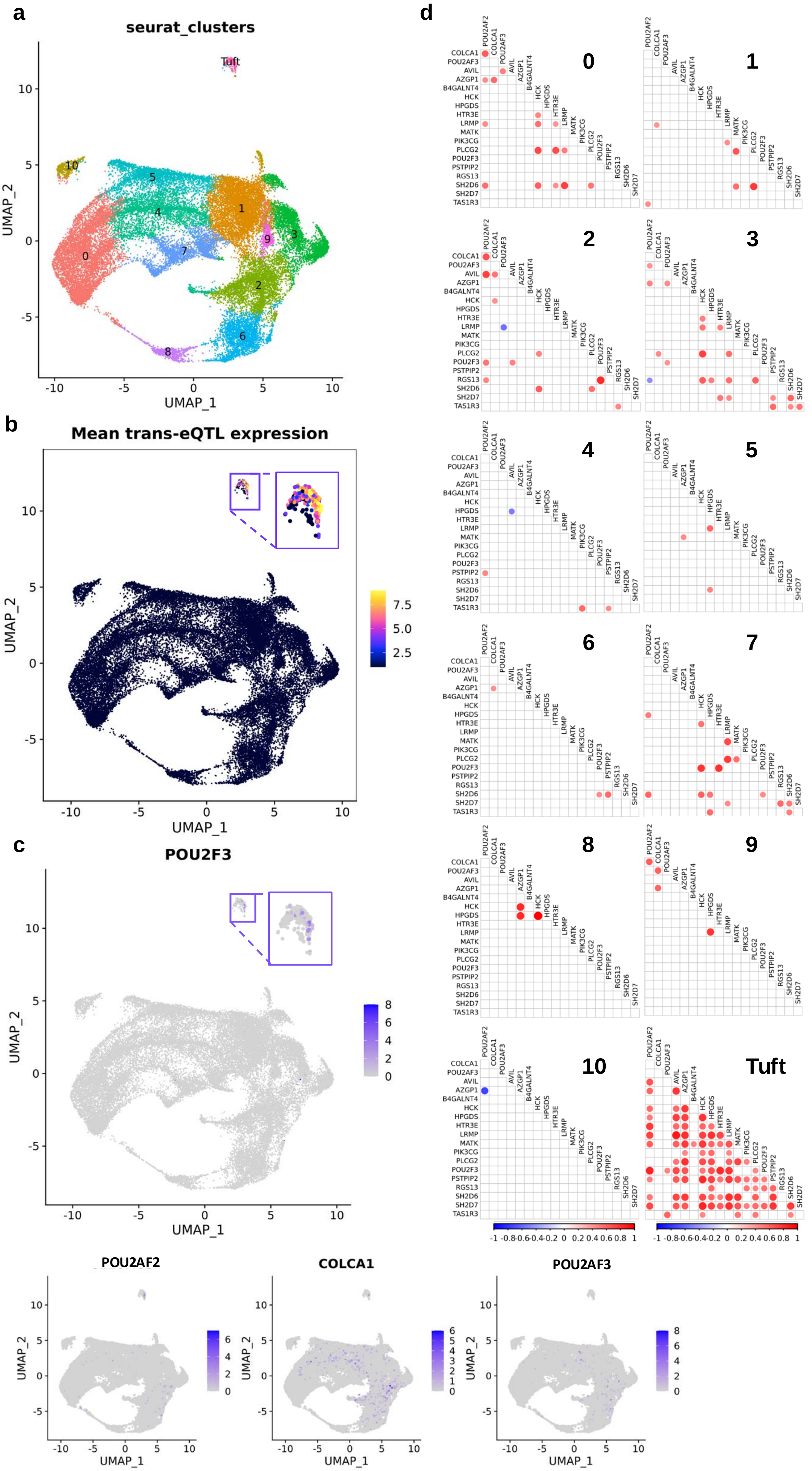

a
seurat_clusters
Tuft
UMAP_1
UMAP_2
b
Mean trans-eQTL expression
c
POU2F3
d
0
1
2
3
4
5
6
7
8
9
10
Tuft
POU2AF2
COLCA1
POU2AF3
AVIL
AZGP1
B4GALNT4
HCK
HPGDS
HTR3E
LRMP
MATK
PIK3CG
PLCG2
POU2F3
PSTPIP2
RGS13
SH2D6
SH2D7
TAS1R3
POU2AF2
COLCA1
POU2AF3

Figure S3. *POU2F3* exhibits tuft cell-specific correlation with *POU2AF2* in the human colon. (a) UMAP embedding of our previous analysis of Smillie *et al.*,[34] healthy human colonic epithelium scRNAseq[7]. (b) The mean expression of refined trans-eQTL targets within cells. (c) *POU2F3* expression across cell, with *POU2AF2, COLCA1 and POU2AF3* for comparison. (d) Pairwise correlations between the expression of refined 11q23.1 trans-eQTL targets across scRNAseq clusters of (a). Only significant ($p<0.05$) associations are shown.
