# Supplementary material for: Genetic variation at 11q23.1 confers colorectal cancer risk by dysregulation of colonic tuft cell transcriptional activator POU2AF2
Source: Gut. 2024 Nov 28;74(5):e332121. doi: 10.1136/gutjnl-2024-332121 (PMC12013567; doi:10.1136/gutjnl-2024-332121)
Supplement: online supplemental file 4 [file gutjnl-74-5-s004.pdf]

a

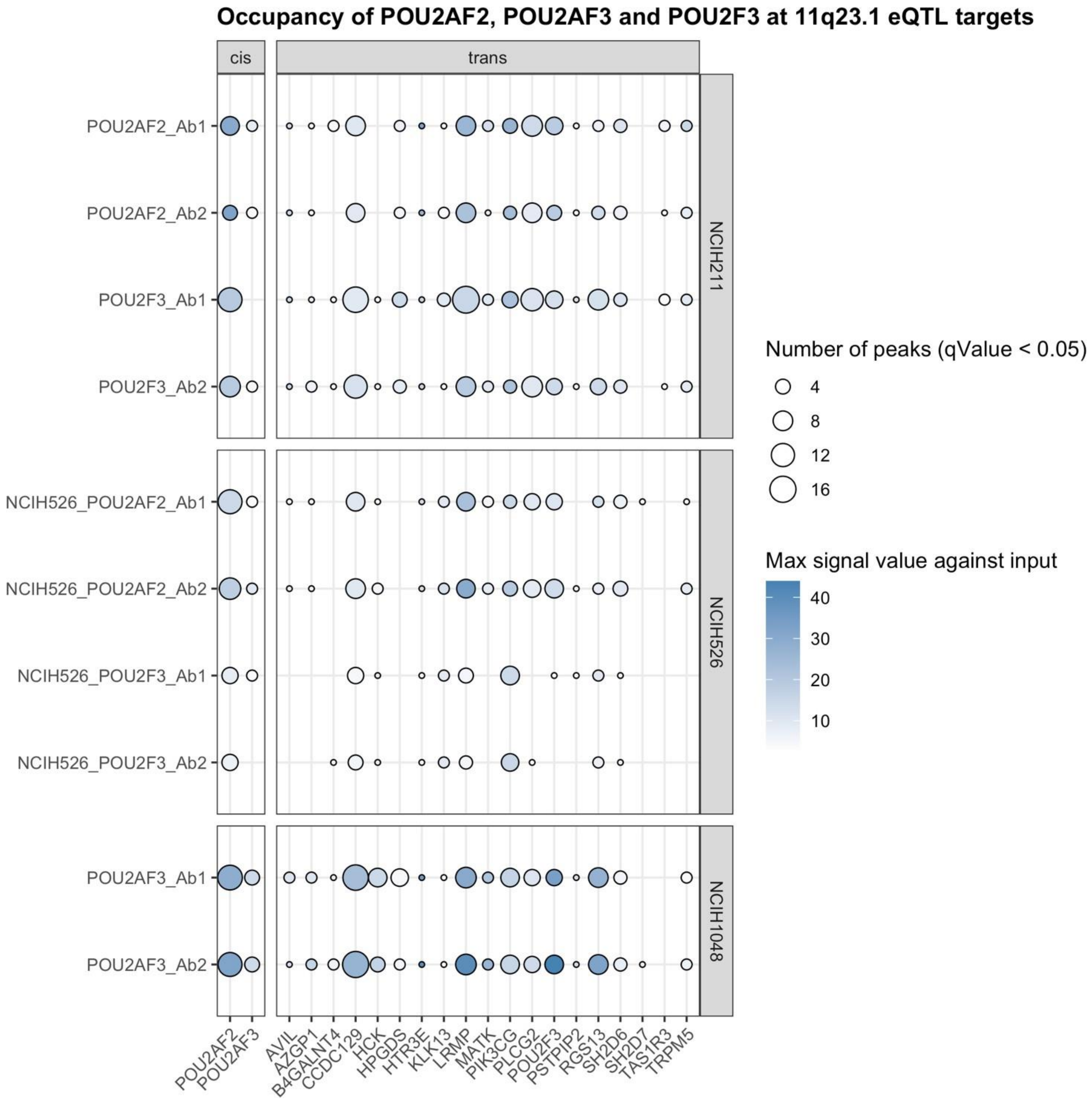
Occupancy of POU2AF2, POU2AF3 and POU2F3 at 11q23.1 eQTL targets
cis
trans
POU2AF2_Ab1
POU2AF2_Ab2
POU2F3_Ab1
POU2F3_Ab2
NCIH211
NCIH526_POU2AF2_Ab1
NCIH526_POU2AF2_Ab2
NCIH526_POU2F3_Ab1
NCIH526_POU2F3_Ab2
NCIH526
POU2AF3_Ab1
POU2AF3_Ab2
NCIH1048
POU2AF2
POU2AF3
AVIL
AZGP1
B4GALNT4
CCDC129
HCK
HPGDS
HTR3E
KLK13
LRMP
MATK
PIK3CG
PLCG2
POU2F3
PSTPIP2
RGS13
SH2D6
SH2D7
TAS1R3
TRPM5
Number of peaks (qValue < 0.05)
4
8
12
16
Max signal value against input
40
30
20
10

b

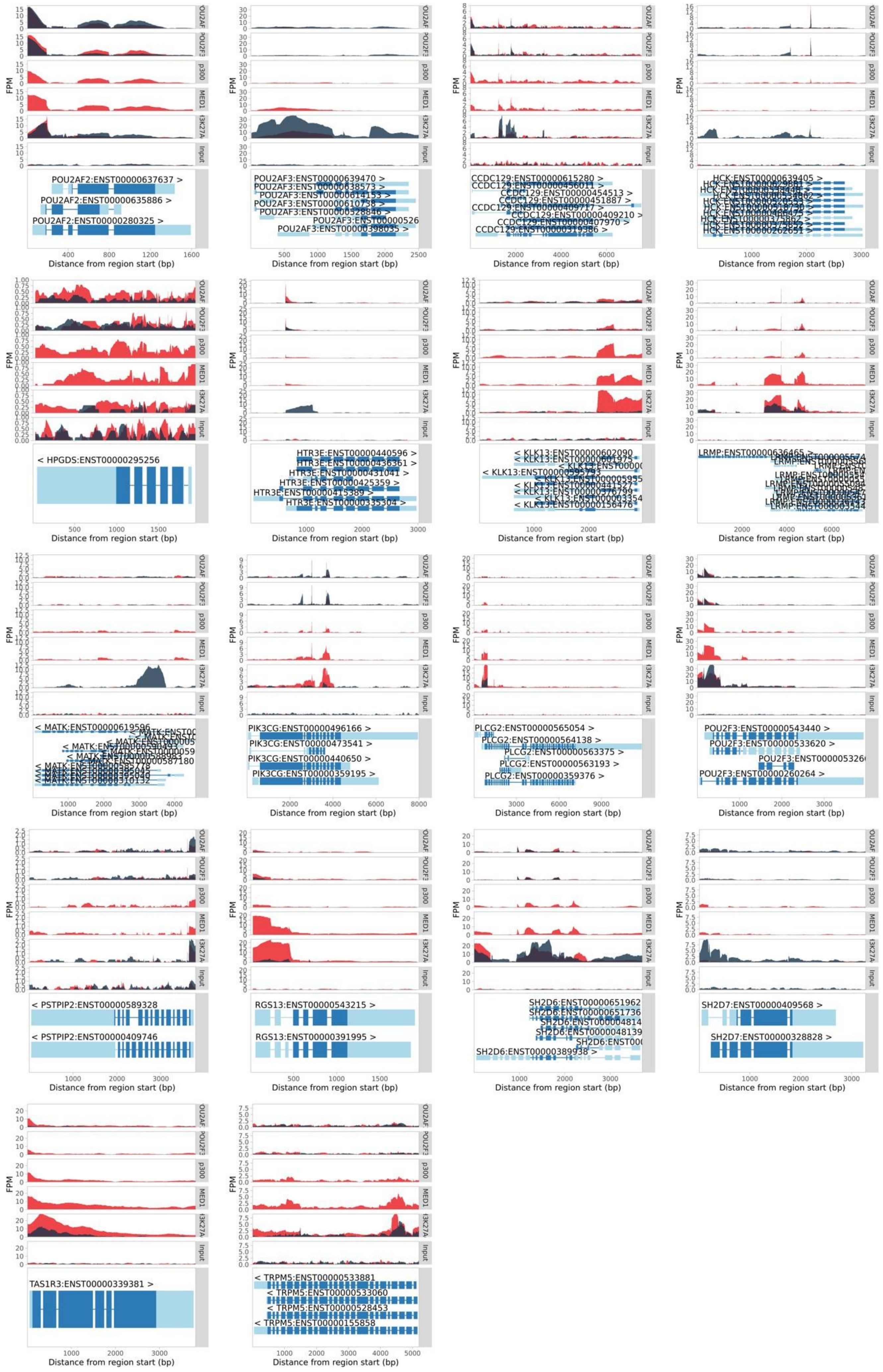

c

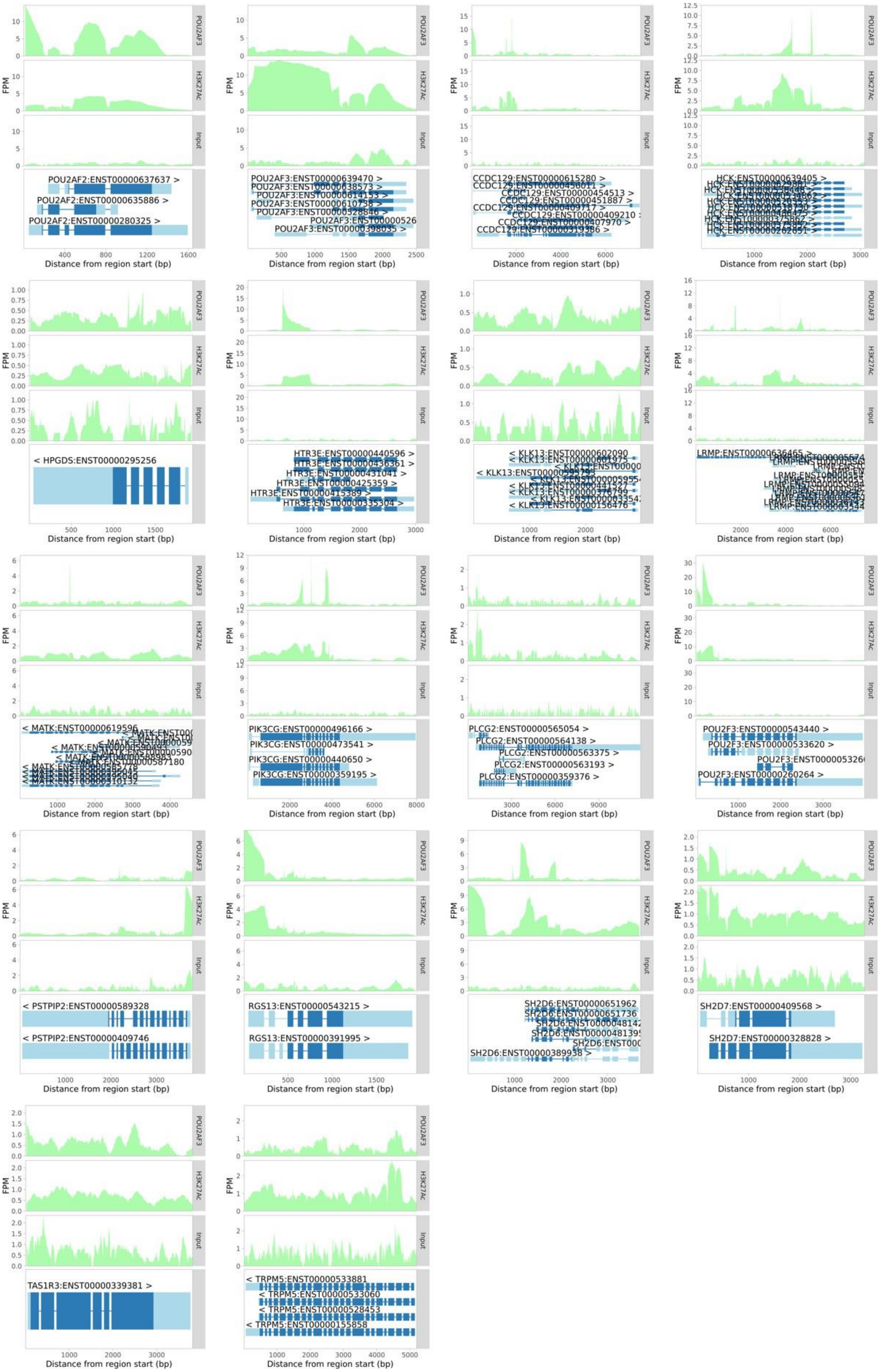

FPM
POU2AF3
H3K27Ac
Input
Distance from region start (bp)
POU2AF2:ENST00000637637 >
POU2AF2:ENST00000635886 >
POU2AF2:ENST00000280325 >
POU2AF3:ENST00000639470 >
POU2AF3:ENST00000398035 >
CCDC129:ENST00000615280 >
CCDC129:ENST00000454513 >
CCDC129:ENST00000451887 >
HCK:ENST00000639405 >
< HPGDS:ENST00000295256
HTR3E:ENST00000440596 >
HTR3E:ENST00000436361 >
HTR3E:ENST00000431041 >
HTR3E:ENST00000425359 >
HTR3E:ENST00000335304 >
< KLK13:ENST00000602090
< KLK13:ENST00000156476
< MATK:ENST00000619596
< MATK:ENST00000310132
PIK3CG:ENST00000496166 >
PIK3CG:ENST00000473541 >
PIK3CG:ENST00000440650 >
PIK3CG:ENST00000359195 >
PLCG2:ENST00000565054 >
PLCG2:ENST00000564138 >
PLCG2:ENST00000563375 >
PLCG2:ENST00000563193 >
PLCG2:ENST00000359376 >
POU2F3:ENST00000543440 >
POU2F3:ENST00000533620 >
POU2F3:ENST00000260264 >
< PSTPIP2:ENST00000589328
< PSTPIP2:ENST00000409746
RGS13:ENST00000543215 >
RGS13:ENST00000391995 >
SH2D6:ENST00000651962
SH2D6:ENST00000651736
SH2D6:ENST00000389938 >
SH2D7:ENST00000409568 >
SH2D7:ENST00000328828 >
TAS1R3:ENST00000339381 >
< TRPM5:ENST00000533881
< TRPM5:ENST00000533060
< TRPM5:ENST00000528453
< TRPM5:ENST00000155858

Figure S4. POU2AF2, POU2AF3 and POU2F3 exhibit correlated binding at 11q23.1 trans-eQTL targets in SCLC cell lines indicative of transcriptional activation. (a) The maximum signal value (enrichment over input) and total number of significant peaks (qvalue < 0.05) where 11q23.1 cis- and trans-eQTL targets were the closest gene in POU2AF2, POU2AF3 and POU2F3 ChIPseq experiments. POU2AF2 (b) and POU2F3 (c) enriched reads at *POU2AF2, POU2AF3* and 11q23.1 trans-eQTL targets bound by core motif sequence in Figure 4c. POU2AF2 traces are merged across antibody replicates. All other proteins were targeted with a single antibody within each cell-line. Red histogram=NCIH211, Grey histogram=NCIH526, green histogram=NCIH1048.
