# Supplementary material for: Genetic variation at 11q23.1 confers colorectal cancer risk by dysregulation of colonic tuft cell transcriptional activator POU2AF2
Source: Gut. 2024 Nov 28;74(5):e332121. doi: 10.1136/gutjnl-2024-332121 (PMC12013567; doi:10.1136/gutjnl-2024-332121)
Supplement: online supplemental file 5 [file gutjnl-74-5-s005.pdf]

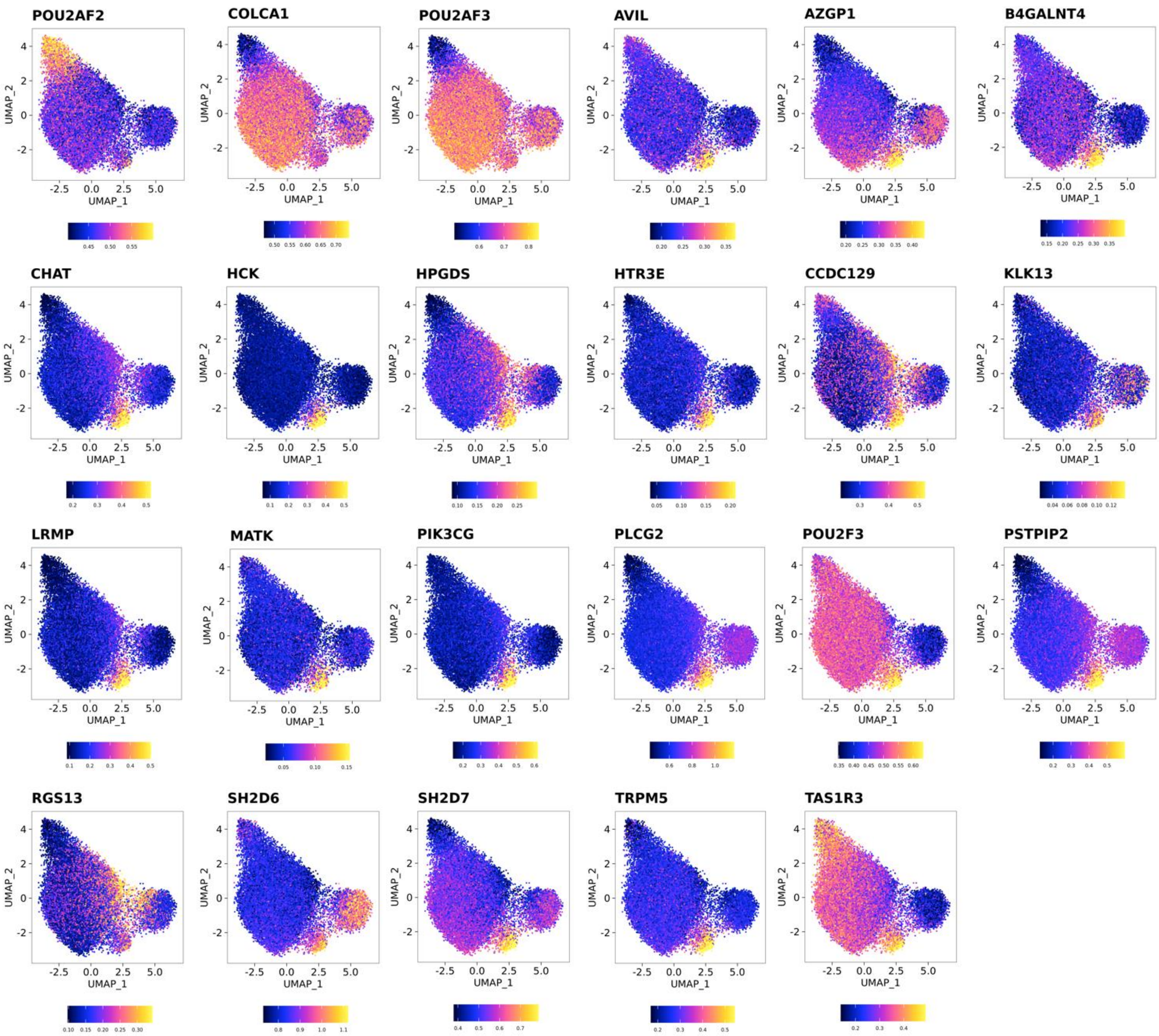

Figure S5. Chromatin at 11q23.1 trans-eQTL targets is exclusively accessible in tuft cells. Normalised accessibility of 11q23.1 cis- and refined trans-eQTL targets across individual cells.
