# Supplementary material for: Genetic variation at 11q23.1 confers colorectal cancer risk by dysregulation of colonic tuft cell transcriptional activator POU2AF2
Source: Gut. 2024 Nov 28;74(5):e332121. doi: 10.1136/gutjnl-2024-332121 (PMC12013567; doi:10.1136/gutjnl-2024-332121)
Supplement: online supplemental file 6 [file gutjnl-74-5-s006.pdf]

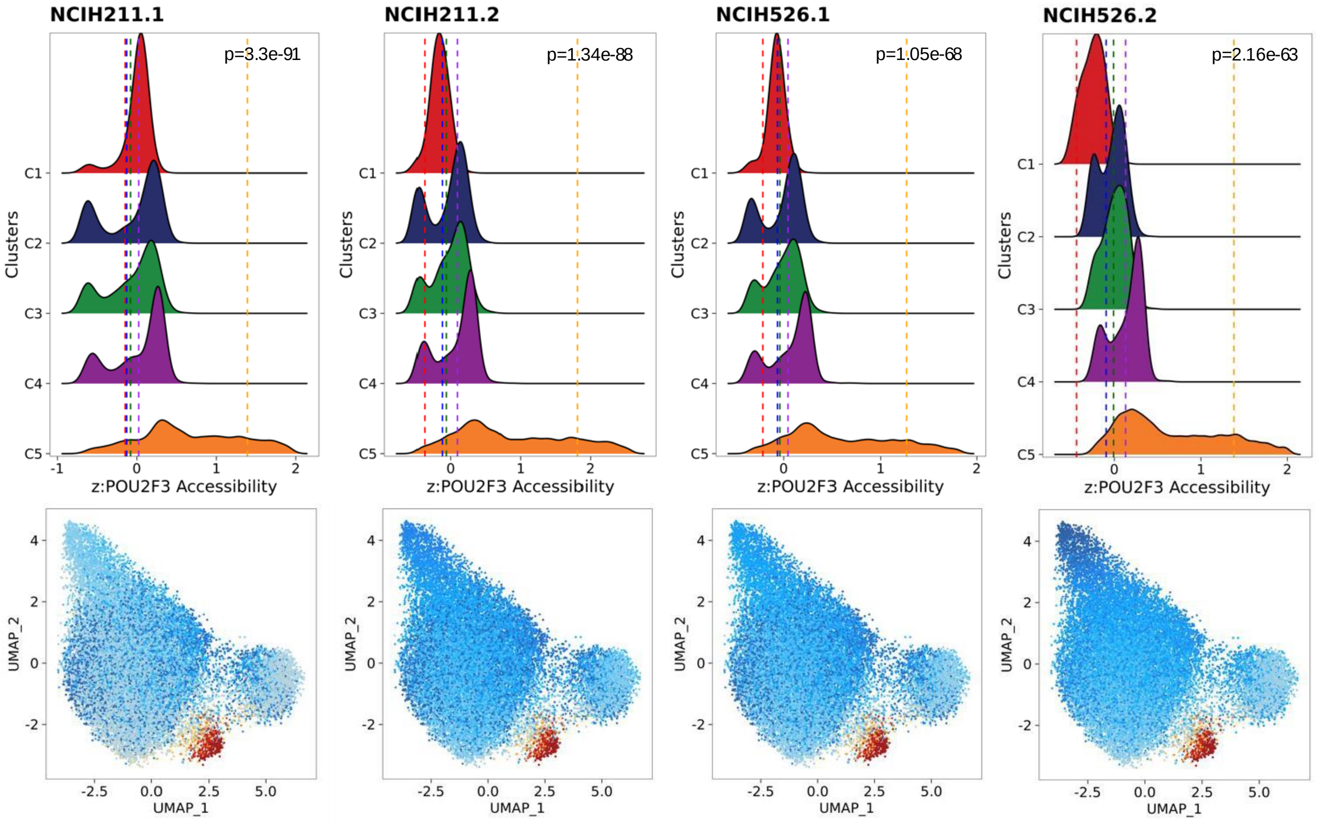

Figure S6. POU2F3-bound sequence accessibility is greatest in tuft-like cells. Relative accessibility of POU2F3-bound sequence accessibility across entire scATAC clusters (above) and individual cells (below). P-values calculated by t-test of normalised enrichment scores in cluster 5 compared to all other clusters combined.
