# Supplementary material for: Genetic variation at 11q23.1 confers colorectal cancer risk by dysregulation of colonic tuft cell transcriptional activator POU2AF2
Source: Gut. 2024 Nov 28;74(5):e332121. doi: 10.1136/gutjnl-2024-332121 (PMC12013567; doi:10.1136/gutjnl-2024-332121)
Supplement: online supplemental file 7 [file gutjnl-74-5-s007.pdf]

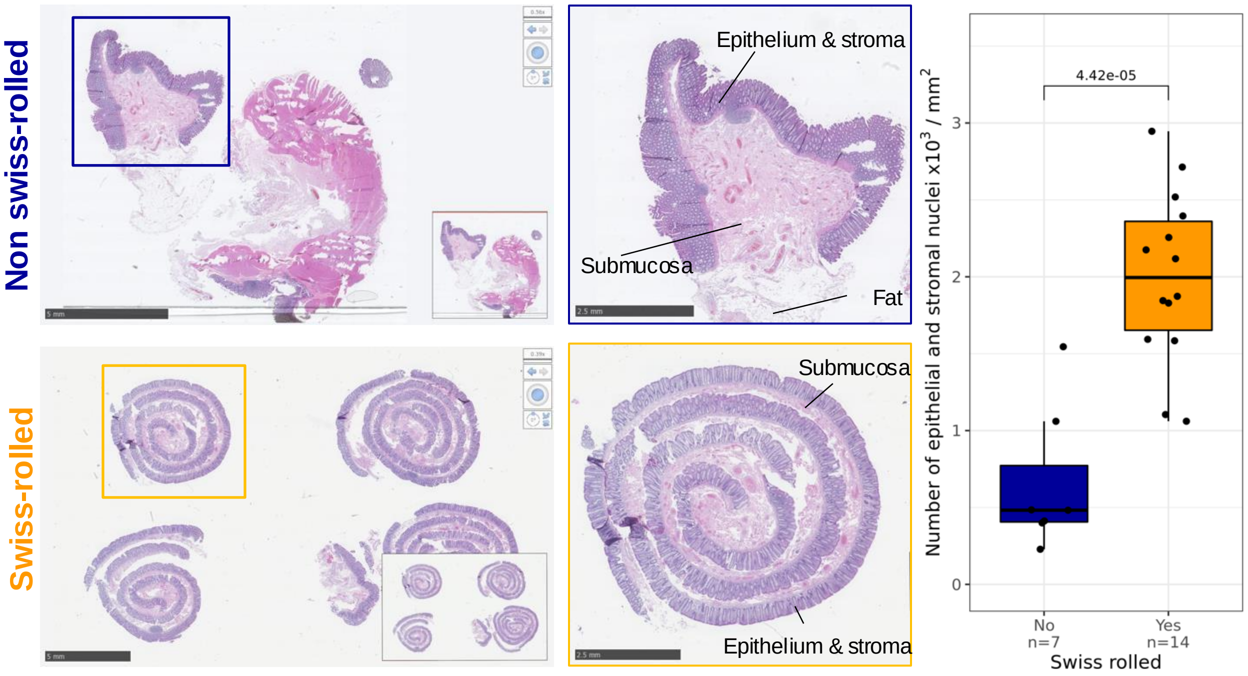

Figure S7. Optimisation of epithelial cell collection for detection of rare cell types. Haematoxylin and eosin stained samples of healthy colonic epithelium pre- (top) and post- (bottom) optimisation of 'swiss-rolling' method. Images taken using NDP View 2. P-value calculated by unpaired t-test.
