# Supplementary material for: Genetic variation at 11q23.1 confers colorectal cancer risk by dysregulation of colonic tuft cell transcriptional activator POU2AF2
Source: Gut. 2024 Nov 28;74(5):e332121. doi: 10.1136/gutjnl-2024-332121 (PMC12013567; doi:10.1136/gutjnl-2024-332121)
Supplement: online supplemental file 8 [file gutjnl-74-5-s008.pdf]

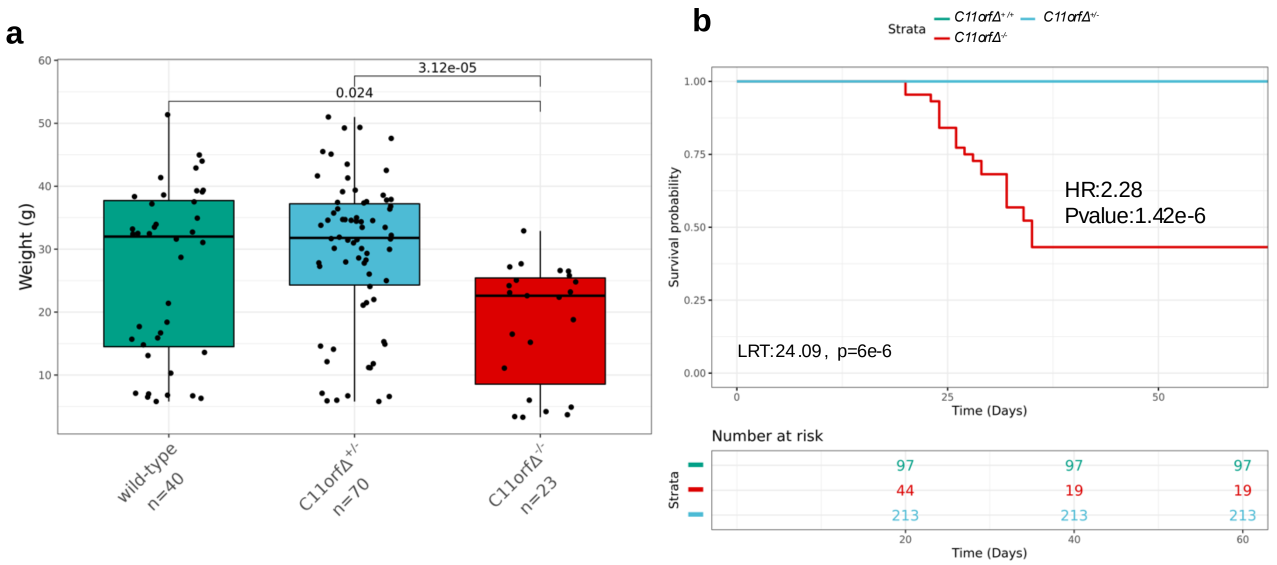

Figure S8. *C11orfΔ*$^{-/-}$ mice exhibit reduced weight and overall survival. Weight (a) and survival (b) of *C11orfΔ* mice. P values for weight comparisons are calculated by Benjamini-Hochberg correction of unpaired Wilcoxon ranks sum tests. Survival P-value calculated by cox proportional hazards model. LRT=Likelihood ration test, HR=Hazard Ratio.
