# Supplementary material for: Genetic variation at 11q23.1 confers colorectal cancer risk by dysregulation of colonic tuft cell transcriptional activator POU2AF2
Source: Gut. 2024 Nov 28;74(5):e332121. doi: 10.1136/gutjnl-2024-332121 (PMC12013567; doi:10.1136/gutjnl-2024-332121)
Supplement: online supplemental file 9 [file gutjnl-74-5-s009.pdf]

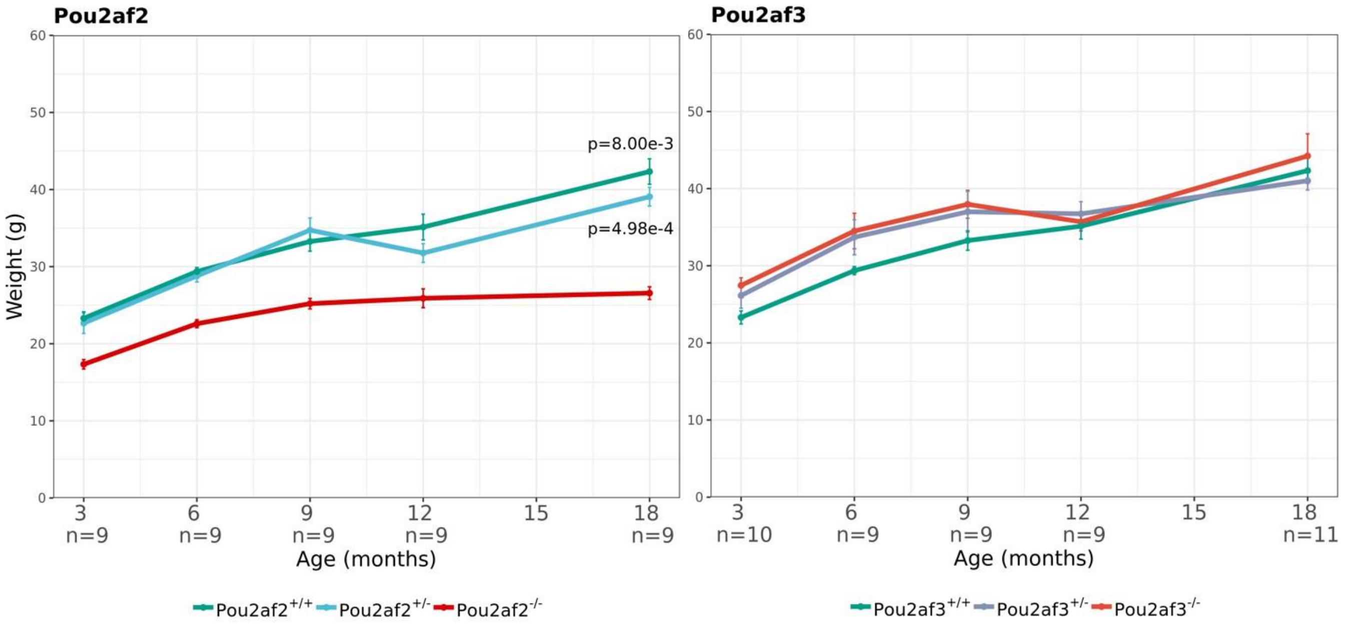

Supplementary Figure S9. ***Pou2af2***$^{-/-}$ **but not** ***Pou2af3***$^{-/-}$ **mice exhibit reduced weight.** Error bars represent standard error about the mean. P-values are calculated by t-test of area under curve statistics for genotype comparisons against *Pou2af2*$^{-/-}$. *Pou2af3* genotype was not associated with any change in weight.
